# Low-Load Unilateral and Bilateral Resistance Training to Restore Lower Limb Function in the Early Rehabilitation After Total Knee Arthroplasty: A Randomized Active-Controlled Clinical Trial

**DOI:** 10.3389/fmed.2021.628021

**Published:** 2021-06-22

**Authors:** Robert Jacksteit, Tino Stöckel, Martin Behrens, Frank Feldhege, Philipp Bergschmidt, Rainer Bader, Wolfram Mittelmeier, Ralf Skripitz, Anett Mau-Moeller

**Affiliations:** ^1^Department of Orthopaedics, University Medicine Rostock, Rostock, Germany; ^2^Institute of Sport Science, University of Rostock, Rostock, Germany; ^3^Department of Sport Science, Otto-von-Guericke University Magdeburg, Magdeburg, Germany; ^4^Department of Traumatology, Orthopaedics and Hand Surgery, Klinikum Südstadt, Rostock, Germany; ^5^Department of Orthopaedics, Roland Klinik, Bremen, Germany

**Keywords:** cross education, strength training, interlimb transfer, continuous passive motion, controlled active motion, range of motion

## Abstract

**Background:** Continuous passive motion (CPM) is frequently used during rehabilitation following total knee arthroplasty (TKA). Low-load resistance training (LLRT) using continuous active motion (CAM) devices is a promising alternative. We investigated the effectiveness of CPM compared to LLRT using the affected leg (CAMuni) and both legs (CAMbi) in the early post-operative rehabilitation. Hypotheses: (I) LLRT (CAMuni and CAMbi) is superior to CPM, (II) additional training of the unaffected leg (CAMbi) is more effective than unilateral training (CAMuni).

**Materials and Methods:** Eighty-five TKA patients were randomly assigned to three groups, respectively: (i) unilateral CPM of the operated leg; (ii) unilateral CAM of the operated leg (CAMuni); (iii) bilateral alternating CAM (CAMbi). Patients were assessed 1 day before TKA (pre-test), 1 day before discharge (post-test), and 3 months post-operatively (follow-up). Primary outcome: active knee flexion range of motion (ROM_Flex_). Secondary outcomes: active knee extension ROM (ROM_Ext_), swelling, pain, C-reactive protein, quality of life (Qol), physical activity, timed-up-and-go performance, stair-climbing performance, quadriceps muscle strength. Analyses of covariances were performed (modified intention-to-treat and per-protocol).

**Results:** Hypothesis I: Primary outcome: CAMbi resulted in a higher ROM_Flex_ of 9.0° (95%CI −18.03–0.04°, *d* = 0.76) and 6.3° (95%CI −14.31–0.99°, *d* = 0.61) compared to CPM at post-test and follow-up, respectively. Secondary outcomes: At post-test, C-reactive protein was lower in both CAM groups compared with CPM. Knee pain was lower in CAMuni compared to CPM. Improved ROM_Ext_, reduced swelling, better stair-climbing and timed-up-and-go performance were observed for CAMbi compared to CPM. At follow-up, both CAM groups reported higher Qol and CAMbi showed a better timed-up-and-go performance. Hypothesis II: Primary outcome: CAMbi resulted in a higher knee ROM_Flex_ of 6.5° (95%CI −2.16–15.21°, *d* = 0.56) compared to CAMuni at post-test. Secondary outcomes: At post-test, improved ROM_Ext_, reduced swelling, and better timed-up-and-go performance were observed in CAMbi compared to CAMuni.

**Conclusions:** Additional LLRT of the unaffected leg (CAMbi) seems to be more effective for recovery of function than training of the affected leg only (CAMuni), which may be mediated by positive transfer effects from the unaffected to the affected limb (cross education) and/or preserved neuromuscular function of the trained, unaffected leg.

**Trial Registration:** ClinicalTrials.gov Identifier: NCT02062138.

## Introduction

Knee osteoarthrosis and total knee arthroplasty (TKA) are accompanied by modulations of sensory feedback due to damage to joint afferents and removal of joint structures as well as knee joint swelling, pain, inflammation, and joint laxity, which leads to structural and functional changes in the nervous system and muscle (1). These impairments contribute to an increased loss of muscle strength and function that limits the performance during activities of daily living (ADL) such as stair climbing, chair rising, and walking (2). A conservative treatment of knee osteoarthrosis may have positive effects on delay and/or avoidance of TKA (3). However, in end stage knee osteoarthrosis, TKA is considered the most effective treatment to reduce pain and restore function (4).

The early restoration of knee joint range of motion (ROM) and physical function are the major objectives after TKA. Continuous passive motion (CPM) is often used during post-operative rehabilitation to improve ROM. However, its effects on physical function of TKA patients are controversially discussed, as a Cochrane review by Harvey et al. concluded that a CPM treatment has no clinically important effects on ROM, pain, function, and quality of life (5).

Low-load resistance training using controlled active motion (CAM) devices may be a promising alternative to CPM treatment. Devries et al. have shown that resistance training with low intensity (30% of maximal voluntary contraction) during a 2-week period of step-reduction (<1,500 steps/day) enhanced muscle anabolic sensitivity in older men. This result indicates that low-load resistance training is an appropriate strategy to preserve muscle mass and function during a phase of reduced physical activity that also affects TKA patients in the early post-operative phase (6). However, only three studies have investigated the effectiveness of CAM treatments compared to CPM in TKA patients (7–9). Two studies compared the effect of a (i) CAM treatment with a slider board (2 ×10 min for 5–7 days) plus physiotherapy, (ii) CPM treatment (3 ×2 h for 5–7 days) plus physiotherapy, and (iii) physiotherapy alone with respect to post-operative ROM in the early rehabilitation after TKA. The authors found no differences between groups in active ROM indicating that the CAM treatment had no beneficial effects for recovery compared to the other treatments (7, 8). However, one study analyzed the effect of a daily (i) CAM treatment using sling exercises (2 ×30 min for 10.0 days) plus physiotherapy and (ii) CPM-treatment (2 ×30 min for 10.5 days) plus physiotherapy on clinical and functional outcomes (9). The results indicated that the CAM treatment (sling exercise intervention plus physiotherapy) had a clinically relevant beneficial short-term effect on passive knee joint ROM compared to CPM. In conclusion, limited knowledge exists about the effects of CAM treatments on clinical and functional outcomes after TKA. The existing evidence does not justify the use of CAM treatments in clinical settings yet. Therefore, the present study aimed at analyzing possible functional and clinical benefits of low-load resistance training using a CAM device in the early rehabilitation after TKA and compared (i) standard CPM treatment, (ii) unilateral CAM treatment (CAMuni), and (iii) bilateral alternating CAM treatment (CAMbi).

The CAMbi intervention aimed to optimize CAM treatment by the attempt to benefit from the positive effects associated with the use of the unaffected leg during rehabilitation after TKA. The phenomenon behind this approach is known as cross education. Cross education is defined as the performance gain (i.e., transfer of strength and skills) in the untrained homologous muscle group after unilateral motor training, which has been shown for a wide range of motor tasks. Different changes within the nervous, muscle, and endocrine systems can explain the processes underlying the transfer of effects from training of the unaffected to the affected leg (10–12). Patients with TKA usually develop interlimb asymmetries (decrements in muscle size, strength, and voluntary activation) as a consequence of decreased mobilization before surgery and especially in the early post-operative phase (1, 13). A review discussing the application of cross education during immobilization reported preservative short-term effects of unilateral training on muscle function of the immobilized, untrained leg (14) indicating cross education as a potential therapeutic approach for restoring limb symmetry and in turn improving recovery of function after TKA (15). However, the literature on how to benefit from cross education effects in orthopedic population is limited (16–20). The effects of cross education have been investigated in patients with knee osteoarthrosis (20), after anterior cruciate ligament (ACL) reconstruction (17–19), and distal radius fracture (16). The clinical utility of cross education to restore function in the early post-operative phase after TKA has not yet been analyzed and is the subject of present study.

We hypothesized that (I) progressive low-load resistance training (CAMuni and CAMbi treatments) would increase maximal active knee flexion ROM (primary outcome), maximal active knee extension ROM, physical activity, quality of life, timed-up-and-go performance, stair-climbing performance as well as quadriceps muscle strength and reduce knee joint swelling, knee pain, and inflammation (C-reactive protein, CRP) to a greater extent than standard CPM treatment. Furthermore, we assumed that (II) positive transfer effects from the unaffected to the affected limb (cross education) during the CAMbi treatment would further benefit early rehabilitation after TKA as compared to CPM and CAMuni treatment.

## Materials and Methods

### Participants

A total of 85 patients scheduled for primary TKA due to clinical and radiological diagnosed severe knee osteoarthrosis were included in this dual-center, three-armed, parallel-group, randomized, active-controlled, double-blinded (investigator, outcome assessor) clinical superiority study (local ethical-vote: A2013-0032). The study was registered at ClinicalTrials.gov (Identifier: NCT02062138) and followed the Consort Guidelines.

This study was conducted at the Department of Orthopedics (Rostock University Medical Center; hospital 1) and the Department of Traumatology, Orthopedics and Hand Surgery (Klinikum Südstadt, Rostock; hospital 2).

Patients were identified as suitable for the study if they were between 50 and 80 years old and had a body mass index (BMI) of <40 kg·m^−2^. Patients with total knee or hip endoprosthesis on the contralateral side were excluded if the surgery was performed within the preceding year. Additional exclusion criteria were: Mini-Mental State Examination score <25, musculoskeletal and neurological disorders that limit physical function, metabolic bone disease, a surgery planned within the next 12 months, and pain or functional restrictions, which would prevent patients from taking part in examinations. Written informed consent was obtained from all patients before participation.

### Surgical Procedure and Pain Management

All participants underwent the same standard surgical procedure involving inserting a non-constrained bicondylar surface replacement system (hospital 1: e.motion®, B|Braun Melsungen AG, Melsungen, Germany; hospital 2: Gemini® SL®, Waldemar Link GmbH & Co. KG, Hamburg, Germany). The surgery was performed by six orthopedic surgeons with an identical surgical approach (Payr's approach). The implants were non-constrained bicondylar surface replacement systems consisting of cemented metallic femoral and tibial components and ultra-high molecular weight polyethylene liners. Smoothening of the lateral patella facet, denervation and soft-tissue balancing were carried out until perfect positioning of the implant components was achieved with respect to biomechanical aspects. Both femoral and tibial components were fixed using PMMA cement (Refobacin Plus Bone Cement, Biomet Deutschland GmbH, Berlin, Germany).

In the early post-operative phase, all participants received a pain-adapted medical analgesia including piritramide (7.5–15.0 mg), metamizole (500.0 mg), and ibuprofen (600.0 mg). Instead of piritramide, the patients in hospital 2 were treated with oxycodone (5.0–10.0 mg). Epidural analgesia or femoral nerve block was prescribed when considered necessary. Patients were discharged from hospital if they were sufficiently mobile (i.e., at least 90° passive knee flexion and no need for personal care) and medically stable.

### Randomization and Blinding

Eligible patients were randomly assigned to one of the three treatment groups using a permuted block randomization by computer-generated tables of random numbers (permuted blocks of variable size; allocation ratio of 1:1:1). Participants were sequentially allocated to the treatments in the order in which they were recruited. After the enrolled patients completed all baseline measurements, intervention assignment were ascertained using sealed, opaque envelopes with consecutive numbering. The investigator who opened the envelopes and carried out the implementation of assignments was not involved in the generation and allocation concealment.

The investigator and outcome assessor were blinded to the intervention. Participants were unaware of the treatment allocation at pre-test. Due to the nature of the intervention, participants and physiotherapists were not blinded during the intervention, at post-test, and follow-up.

### Study Interventions

The participants were randomly allocated to one of three treatment groups:

- continuous passive motion (CPM) unilateral operated leg (active control group; standard-of-care therapy)
- continuous active motion unilateral operated leg (CAMuni)
- continuous active motion bilateral alternating (CAMbi).

CPM and CAM interventions were conducted from the second to the ninth post-operative day during hospital stay. Maximal knee flexion and extension ROM was gradually increased according to the patient's tolerance and pain.

#### Continuous Passive Motion Treatment (CPM)

Treatment group I received three CPM interventions per day for 30 min each, using a Kinetec® OptimaTM S3 (AbilityOne Kinetec S. A., Tournes, France). The foot of the operated leg was fixed to the device with a belt and the knee joint was passively moved through a controlled ROM (i.e., from full extension (0°) to maximal tolerated knee flexion) at highest adjustable speed. Participants were instructed not to resist the motion of the device or actively support it.

#### Continuous Active Motion Treatment

**Figure 1:**
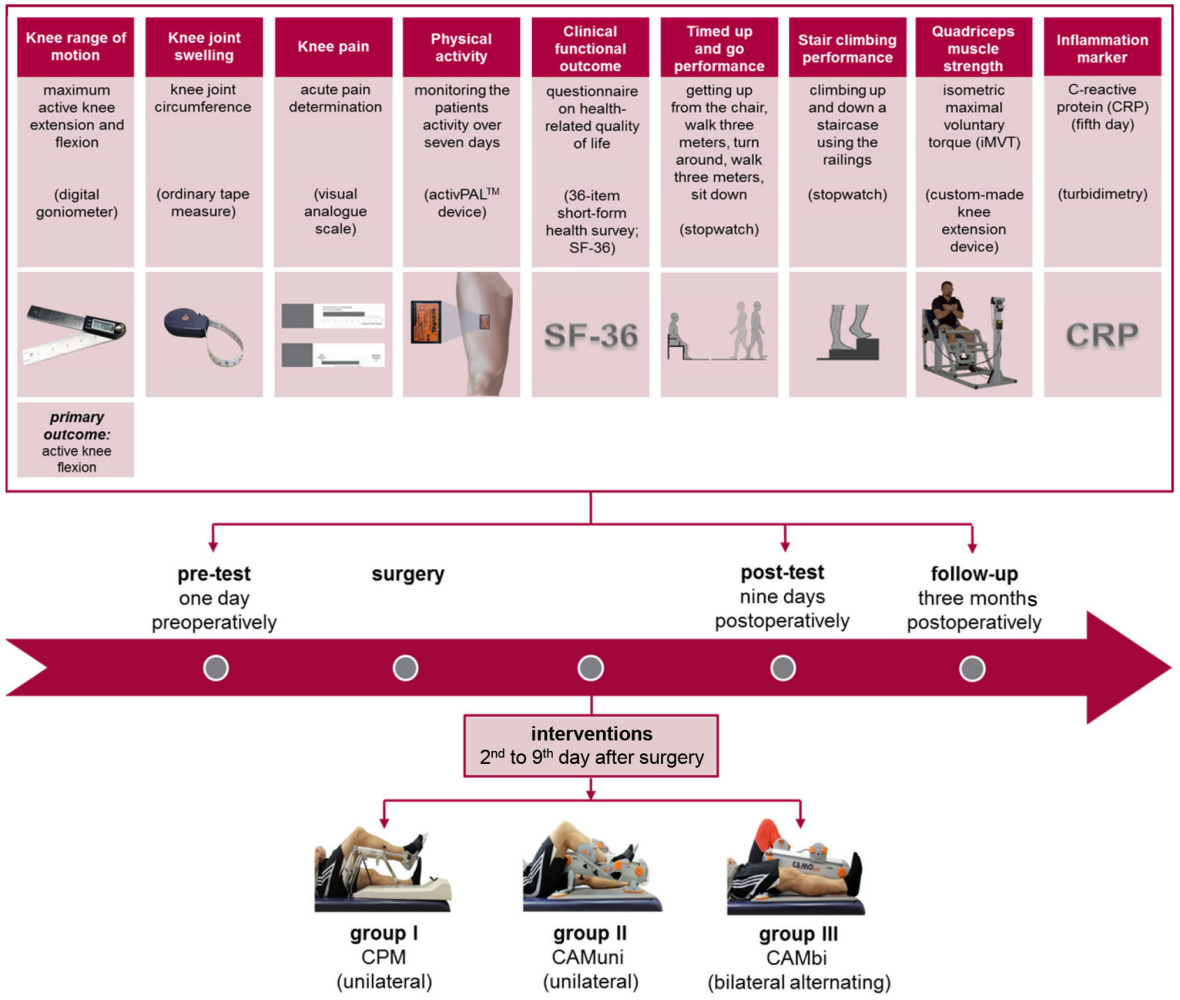
Overview of groups, interventions, measuring points, and relevant primary and secondary outcomes.

The participants of treatment group II (CAMuni) and group III (CAMbi) received a low-load resistance training of the operated leg three times daily for ~30 min with a CAMO®ped device (OPED, Valley, Germany) (Figure 1). The CAMbi group further received a CAM treatment for the non-operated leg for 30 min once a day. During the CAMbi intervention, repetitions were initially performed with the operated leg and afterwards with the other leg.

The leg was fixed to the device with belts at the ankle joint, shinbone, and midfoot. Thus, evasive movements during movement execution were reduced. Both groups performed active knee extensions and flexions through a controlled ROM (i.e., from full extension (0°) to maximal tolerated knee flexion) at self-selected speed. The CAM-treatment was carried out under the supervision of a therapist in order to ensure correct movement execution. The CAM device has four resistance levels (0 = lowest resistance level). The warm-up protocol consisted of one set of 20 repetitions at resistance levels 0 to 2, respectively. Afterwards, five sets at resistance level 3 were performed, each until volitional exhaustion. Between the sets, there was a rest interval of 3 min. The cool down included 20 repetitions at resistance level 0.

The resistance levels of the CAMO®ped device are not individually adjustable. To estimate exercise intensity during CAM treatment, myoelectric activity of vastus lateralis and rectus femoris of the operated leg was recorded. However, the regular post-test procedure was very extensive and we did not want to impose this additional effort on all study participants. Therefore, muscle activity was measured in only three patients to obtain some information on exercise intensity during CAM treatment. A detailed description of EMG measurement was given previously (21). The patients performed 10 repetitions at resistance level 3. The transitions from knee extension to flexion were measured using an electro goniometer (Biometrics Ltd, Newport, United Kingdom). The EMG signals were rectified and averaged (AEMG). AEMG was normalized to the muscle activity recorded during isometric maximal voluntary torque (iMVT) production (%AEMG_iMVT_). The %AEMG_iMVT_ of rectus femoris and vastus lateralis was 10.0 and 32.7% during knee extension and 13.3 and 8.4% during knee flexion, respectively, classifying the CAM treatment as a low-load resistance training.

#### Standardized Inpatient and Outpatient Physiotherapy

In addition to CPM or CAM intervention, all patients participated in standardized in-hospital physiotherapy performed by physiotherapists once daily for 25 to 30 min (except Sundays) from the first post-operative day until discharge from hospital. The intensity of physiotherapeutic exercises was gradually increased, depending on the pain and tolerance of the patient. All patients underwent immediate full weight-bearing mobilization (four-point gait with two crutches) from the second post-operative day. The detailed description of the standardized in-hospital physiotherapy is provided in the Supplementary File 1 (Tables S1–S3).

After discharge from the hospital, patients were treated in an outpatient or inpatient rehabilitation center for 3 weeks. Patients taking part in outpatient rehabilitation stayed at home and attended daily physiotherapy in the nearest rehabilitation center. The inpatient care represents a German peculiarity compared with international standards and was carried out in a rehabilitation clinic. Inpatient and outpatient rehabilitation programmes consisted of daily (except weekends) physiotherapy (individual and/or group therapy), gait training, aqua exercise, bicycle ergometer training, CPM, medical training therapy, manual lymphatic drainage, training courses for patients, physical therapy (incl. sling exercises and training), and traction treatment.

### Assessments and Outcomes

The patients were examined with comprehensive clinical, functional, and strength measurements at three points in time over a period of 3 months:

- 1 day before TKA (pre-test)
- 9 days post-operatively (post-test)
- 3 months after TKA (follow-up).

The primary outcome measure was the active knee flexion ROM. Secondary outcome measures included clinical parameters (active knee extension ROM, knee joint swelling, knee pain, inflammation (CRP), quality of life (SF-36), physical activity (number of steps and sit-to-stand-transitions), and functional outcomes (timed-up-and-go performance, stair-climbing performance, quadriceps muscle strength). A detailed overview of the experimental design is provided in Figure 1.

#### Range of Motion of the Knee Joint

ROM of active knee flexion (primary outcome) and active knee extension were assessed using a commercially available digital long-arm goniometer (300 mm 2 in 1 Electronic Digital Protractor Goniometer Angle Finder Miter Gauge, iGAGING, San Clemente, CA, USA; accuracy: ± 0.20°; repeatability: 0.05°) (22). During the measurement, the patient was positioned in the supine position. The pivot point of the goniometer was aligned with the axis of the knee joint. The arms of the goniometer were aligned with bony anatomical landmarks on proximal (femur) and distal (tibia) body segments, i.e., one arm of the goniometer was aligned parallel to the longitudinal axis of the femur with the reference point trochanter major; and the other arm was located parallel to the longitudinal axis of the tibia with the malleolus lateralis as reference point. The patients actively moved the knee joint throughout its full range of motion, while the investigator held the arms of the goniometer in line with the anatomical landmarks. The maximum active knee extension and flexion joint angles were measured in degrees. Positive knee extension angles mean that full knee extension ROM of 0° was not reached (ROM deficit).

In order to ensure the formal intra-rater reliability within the same session (intra-session), the intraclass correlation coefficient (ICC) and coefficient of variation (CV) for maximal active knee flexion and active knee extension ROM were calculated for 11 gonarthrosis patients. The results showed high absolute (knee flexion CV = 0.81%; knee extension CV = 0.83%) and relative (ICC's = 0.99) intra-rater reliability.

#### Knee Pain

A visual analog scale was used to assess acute knee pain after TKA implantation (23). The participants laid relaxed in supine position and were asked to mark their perceived knee pain on a horizontal scale by using a slider (100 mm). The two endpoints of the scale represent the extremes “no pain” (left end; happy face) and “intolerable pain” (right end; unhappy face). The quantification of pain was performed by a millimeter scale (from 0 - 100 mm) on the back of the measuring instrument (“0” indicated “no pain” and “100” indicated “intolerable pain”). High reliability has been demonstrated for acute pain measurements when using the visual analog scale (ICC = 0.97) (23).

#### Knee Joint Swelling

Knee joint circumference was assessed using an ordinary tape measure (hoechstmass®, Sulzbach, Germany). During the examination, the patient was in a supine position with the knee joint in full extension. The measurement of circumference was performed 1 cm above the superior border of the patella (24).

Intra-rater reliability within the same session (intra-session) of the measurement was determined for 11 patients with gonarthrosis. The results demonstrated a high absolute (CV = 5.89%) and relative (ICC = 0.99) intra-rater reliability, which is in line with other studies reporting high intra- and inter-session reliability for circumferential measurements in TKA patients (ICC values between 0.98 and 0.99) (24).

#### Physical Activity

Physical activity of the patients was recorded with an activity detection system (PAL Technologies Ltd., Glasgow, UK) (25). The inclination of the femur was measured by means of an accelerometer. The wireless sensor (53 mm in length, 35 mm in width, and 7 mm in depth) was attached anteriorly in the middle of the thigh of the unaffected leg with Fixomull™ (BSN medical GmbH, Hamburg, Germany). Physical activity was measured continuously over a period of 7 days, i.e., during hospital stay (second to eighth post-operative day) and 3 months post-operatively (except when performing activities in the water, e.g., taking a shower, swimming). Data were recorded with a sampling frequency of 10 Hz. The total number of steps and sit-to-stand transitions for a 7-day period were calculated using the activPAL^TM^ interface program (version 7.1.18).

Dowd et al. compared the activPAL^TM^ device with the ActiGraph device. The authors documented a high validity of the activPAL^TM^ for step count (26). Furthermore, Dahlgren et al. examined step counts over a period of 1 week with regard to inter-session reliability in a healthy population and demonstrated high relative reliability (ICC's > 0.70) for different physical activities (i.e. treadmill walking, self-paced walking, and stair walking) (25).

#### Health-Related Quality of Life (SF-36)

The Short-form (36) Health Survey (SF-36) questionnaire is one of the most frequently used questionnaires for the assessment of the subjective state of health or health-related quality of life (27). The score consists of 36 items assigned to eight dimensions of quality of life (eight subscales): physical functioning, social role functioning, physical role functioning, emotional role functioning, bodily pain, mental health, vitality, general health perceptions. One total score (SF-36 score) as a mean of the eight subscales and two subscores (Mental health and Physical health) was calculated. A high value on a scale of 0 to 100 represents a subjectively perceived good health condition. The SF-36 questionnaire was carried out at pre-test and follow-up.

#### Timed-Up-and-Go Performance

Mobility of the patients was assessed with the timed-up-and go-test (28). The patients had to rise from a chair with armrests, walk a defined distance of 3 m, turn back, and sit down again. The seat height of the chair was 48 cm and the height of the armrests 68 cm. The task should be executed safely and quickly using regular footwear and crutches if required. The time was measured with a stopwatch (Kienzle, Hamburg, Germany). The fastest of the two trials was used for data analysis.

#### Stair-Climbing Performance

The stair-climbing-test is a clinical physical performance measure for estimating postural control and strength of the lower extremities (28). The patients were asked to climb a staircase of eight steps (step height: 17.5 cm) in a safely and quickly manner using a railing and regular footwear. The time was measured using a standard stopwatch (Kienzle, Hamburg, Germany). One trial was performed and analyzed.

#### Isometric Maximal Voluntary Torque

The measurement of iMVT was performed on a custom-made knee extension dynamometer (21, 29). The device allowed an individual positioning of the patients. The examinations were carried out with the affected leg at constant joint angles (hip joint: 90°, ankle joint: 90°, and knee joint: 60–70°; 0° = full extension). During testing, the trunk of the patients was fixed with velcro straps across the waist and the shoulder to reduce excessive movements. The shin was fixed 2–3 cm above the lateral malleolus. Throughout the measurement, the patients folded their arms in front of their chest and were asked to extend the leg isometrically against a panel for 3 s.

The patients were instructed to act as forcefully as possible. An investigator verbally motivated the patients and checked that the contraction was performed without any visible countermovement or pretension. At least three to five familiarization trials were carried out. The patient was familiar with the test procedure if the CV of successive iMVTs was below 5% (CV = standard deviation/mean ×100). The mean value of the three test trials was used as measure of iMVT.

The force signal was captured with a KM40 force sensor (ME-Messsysteme GmbH, Hennigsdorf, Germany), preamplified (GSV3, ME-Messsysteme GmbH, Hennigsdorf, Germany) and recorded with a sampling rate of 3 kHz with the Telemyo 2400T G2 EMG telemetry system. The signals were filtered using MATLAB (version R2012b; The Math-Works, Inc., Natick, MA, USA): third-order Butterworth IIR low-pass filter (25 Hz). Torque was calculated by multiplying the length of the lever arm with the force.

Prior to the study, the intra-rater reliability within the same session (intra-session) of iMVT measurement was assessed in 20 healthy age-homogeneous volunteers (age: 62.1 ± 6.2 years) and 20 patients with knee osteoarthrosis (age: 66.7 ± 8.8 years). The results showed a high relative intra-rater reliability (ICC's = 0.99) and high absolute intra-rater reliability (healthy group: CV = 3.76%; patient group: CV = 5.20%) in both groups.

#### C-Reactive Protein

The level of CRP serves inter alia as a biomarker for inflammation and periprosthetic joint infection (30). Venous blood samples were obtained before surgery and on the fifth post-operative day. The measurements were performed according to the manufacturer's instruction using the standard turbidimetric technique (31).

### Statistical Analysis

Active knee joint ROM was chosen as primary outcome variable as the ROM is a primary indicator for a successful TKA and is required for the performance of ADL (32, 33). A recent Cochrane review by Harvey et al. analyzed the effectiveness of CPM as a supplement therapy to standard physiotherapy in TKA patients (5). To justify the use of the additional CPM therapy, Harvey et al. defined a higher knee flexion ROM of 5° as clinically relevant. Therefore, we have also assumed a difference between CPM and CAM treatments in active knee flexion ROM of at least 5° to be of clinical relevance. However, there are no studies on the comparison of CAM treatment (using the CAMO®ped device) and CPM treatment, making it impossible to adequately calculate sample size on the basis of preliminary results. Thus, we assumed a large effect (Cohen's *f* = 0.40) with a two-sided significance of 0.050 and a power of 0.80 to estimate sample size. According to this, a total of 66 patients (22 patients in each group) were required for the trial. A recruitment period of 24 months was assumed for the enrolment of patients.

The modified intention-to-treat analysis (mITT) included all randomized patients according to their original treatment allocation who started the treatment (*n* = 66), i.e., patients who were randomized but never received any treatment were excluded from the analyses (34).

In addition, a sensitivity analysis for each outcome was performed on a per-protocol (PP) basis to test the robustness of the main analysis, i.e., only those patients were included who completed the treatment originally allocated and participated in pre-, post-test, and follow-up (“completers only”; *n* = 60).

Data were checked for normal distribution using the Kolmogorov-Smirnov-Test. In the mITT analysis, multiple imputations (10 imputed data sets) were used to account for missing data using the Markov Chain Monte Carlo method. Differences between the groups were tested for significance using Fisher's exact test, unpaired Student's *t*-test, Pearson chi-squared test or analysis of covariance (ANCOVA) including all three groups (adjusted for baseline, pain, swelling, age, BMI, sex, and hospital). Holm-Sidak *post-hoc* tests were conducted to determine differences between groups. Statistical values of the ANCOVA (*p, F*, and η_*p*_^2^) were calculated from the log-transformed (Lg10) or reverse-transformed data if data was not normally distributed.

It is recommended to use effects sizes for interpreting results of intervention studies to determine the practical relevance and generalizability of results (35). Thus, partial eta-squared (η_*p*_^2^), Cohen's *f* , and Cohen's *d* were calculated as measures of effect size. The effect size Cohen's *f* was used for ANCOVA and interpreted using the following classification: *f* = 0.10 small effect, *f* = 0.25 medium effect, *f* = 0.40 large effect. Furthermore, Cohen's *d* effect size was used to determine the statistical relevance of mean differences between two groups (effect size for *post-hoc* comparisons) with 0.50 to 0.79 indicating a medium effect and 0.80 or higher a large effect. Pooled multiple imputation data are presented as covariate-adjusted mean values (adjusted standard deviation) together with the adjusted mean difference (adjusted 95% confidence interval, 95% CI) in the tables and figures (36).

Sample size and effect sizes were calculated with the statistical software package G^*^Power (version 3.1.9.). All other analyses were performed using SPSS statistical package 22.0 (SPSS Inc., Chicago, IL, USA).

Furthermore, intra-rater reliability was calculated using an Excel spreadsheet developed by Hopkins (37). The CV was calculated as measure of absolute reliability. A CV value of ≤ 10% was defined as high reliability. Relative reliability was estimated using the ICC. An ICC value ≥ 0.90 was considered high, values between 0.80 and 0.90 as moderate and ≤ 0.80 as low (38).

## Results

### Enrollment and Follow-Up

**Figure 2:**
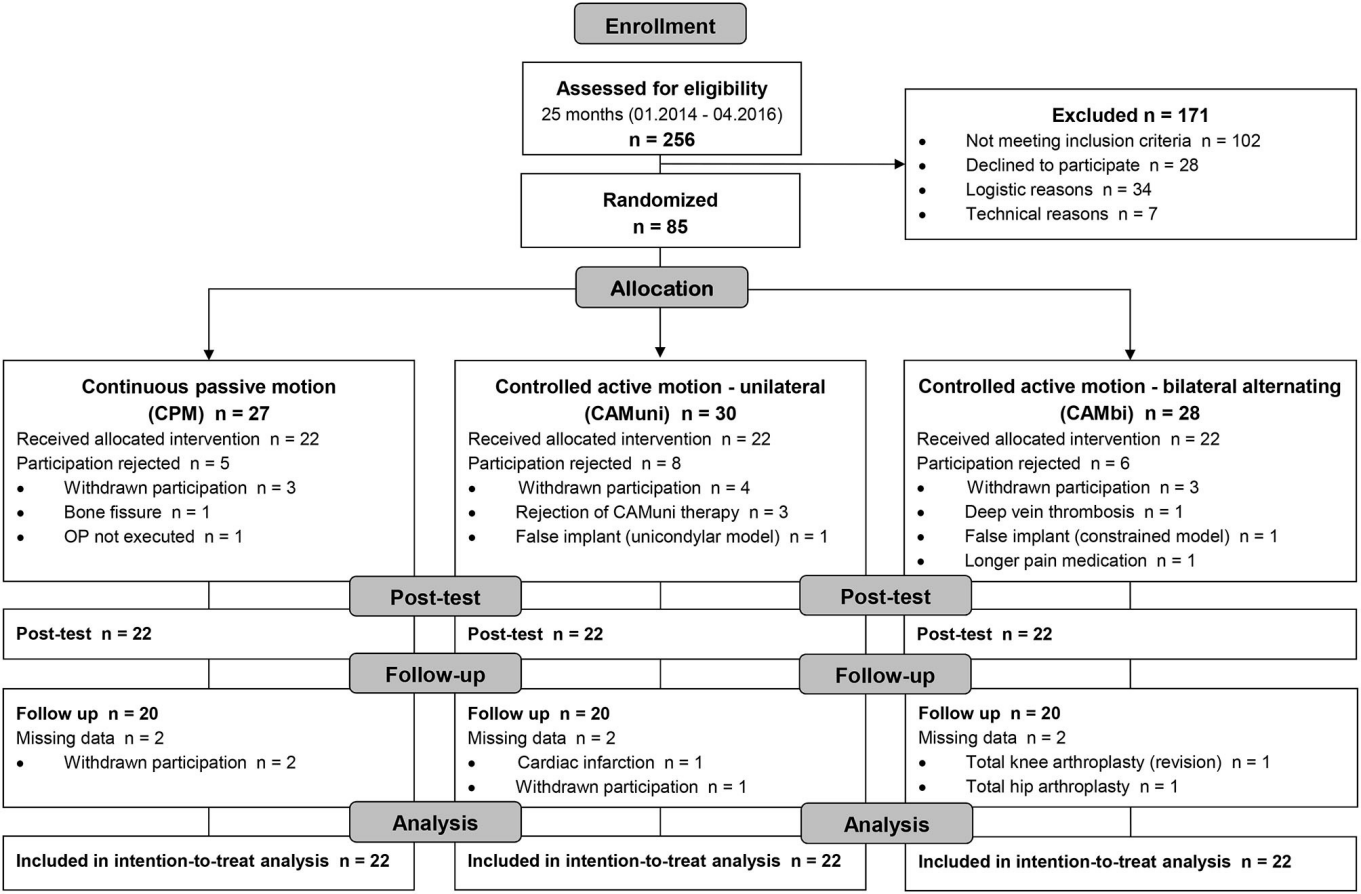
Consort participant flow diagram showing enrollment, intervention allocation, number of participants at different time points, and number of patients included in modified intention-to-treat analysis.

Patient recruitment was stopped when the planned sample size was reached. Eighty-five patients underwent randomization. However, 19 patients were excluded from the full analysis because the treatment was not applied. Thus, 66 patients (22 in each group) received at least one intervention and were included in the mITT analysis. Complete information on the reasons for non-participation in treatment that led to exclusions after randomization is provided in the CONSORT flow diagram (Figure 2). The reasons for post-randomization exclusion were not related to the treatment.

In each group, 20 of 22 patients completed the 3-month follow-up (drop-out rate 9.1% per group). Only patients who completed the originally assigned treatment and participated in the pre-, post-test, and follow-up were included in the PP analysis (*n* = 60).

Enrollment, intervention allocation, number of participants at different time points, and data analysis are reported in the CONSORT flow diagram (Figure 2). No significant differences in the patients' demographic and clinical baseline characteristics were observed (Table 1). The CPM and CAM treatment had no detrimental effects on the patients.

**Table 1:** Demographic and clinical subject characteristics.

	**CPM** ** (*n* = 22)**	**CAMuni** ** (*n* = 22)**	**CAMbi** ** (*n* = 22)**	***p***
**Age (yrs)^‡^**	68.45 (9.07)	67.36 (9.61)	68.55 (8.94)	0.894
**Weight (kg)^‡^**	88.71 (14.44)	89.09 (19,85)	90.42 (12.30)	0.932
**Height (m)^‡^**	1.66 (0.09)	1.66 (0.09)	1.69 (0.09)	0.579
**Body mass index (kg/m**^**2**^**)^‡^**	32.05 (4.68)	32.33 (6.52)	31.81 (4.25)	0.948
**Sex (males)^¥1^**	8.0 (36.4%)	9.0 (40.9%)	8.0 (36.4%)	1.000
**Operated leg (right leg)^¥1^**	13.0 (59.1%)	8.0 (36.4%)	14.0 (63.6%)	0.171
**Mini-Mental state examination^‡^**	28.58 (1.80)	28.80 (1.75)	29.05 (1.75)	0.681
Missing data^§^	3.0 (13.6%)	2 (9.1%)	2 (9.1%)	
**Post-test (d)^‡^**	9.18 (0.66)	8.86 (0.53)	9.23 (0.53)	0.053^†^
**Follow-up (d)^‡^**	88.85 (6.24)	88.60 (7.50)	90.90 (6.87)	0.515
Missing data^§^	2 (9.1%)	2 (9.1%)	2 (9.1%)	
**Comorbidities (ICD-Codes)^¥1^**				
A00–B99	0.0 (0.0%)	1.0 (4.5%)	0.0 (0.0%)	1.000
C00–D48	4.0 (18.2%)	2.0 (9.1%)	3.0 (13.6%)	0.901
D50–D89	2.0 (9.1%)	3.0 (13.6%)	2.0 (9.1%)	1.000
E00–E90	11.0 (50.0%)	14.0 (63.6%)	13.0 (59.1%)	0.742
F00–F99	2.0 (9.1%)	3.0 (13.6%)	1.0 (4.5%)	0.864
G00–G99	2.0 (9.1%)	3.0 (13.6%)	2.0 (9.1%)	1.000
H00–H59	1.0 (4.5%)	1.0 (4.5%)	4.0 (18.2%)	0.348
H60–H95	0.0 (0.0%)	1.0 (4.5%)	1.0 (4.5%)	1.000
I00–I99	17.0 (77.3%)	18.0 (81.8%)	18.0 (81.8%)	1.000
J00–J99	3.0 (13.6%)	4.0 (18.2%)	4.0 (18.2%)	1.000
K00–K93	4.0 (18.2%)	1.0 (4.5%)	4.0 (18.2%)	0.366
M00–M99	16.0 (72.7%)	15.0 (68.2%)	11.0 (50.0%)	0.360
N00–N99	5.0 (22.7%)	4.0 (18.2%)	3.0 (13.6%)	0.920
R00–R99	0.0 (0.0%)	2.0 (9.1%)	1.0 (4.5%)	0.767
Z00–Z99	3.0 (13.6%)	5.0 (22.7%)	7.0 (31.8%)	0.413
**Number of operations per surgeon^¥2^**				0.484
Surgeon 1	10.0 (45.5%)	11.0 (50.0%)	8.0 (36.4%)	
Surgeon 2	6.0 (27.3%)	3.0 (13.6%)	1.0 (4.5%)	
Surgeon 3	2.0 (9.1%)	3.0 (13.6%)	6.0 (27.3%)	
Surgeon 4	0.0 (0.0%)	1.0 (4.5%)	1.0 (4.5%)	
Surgeon 5	4.0 (18.2%)	4.0 (18.2%)	5.0 (22.7%)	
Surgeon 6	0.0 (0.0%)	0.0 (0.0%)	1.0 (4.5%)	
**Number of operations per hospital^¥1^**				1.000
Hospital 1	19.0 (86.4%)	19.0 (86.4%)	19.0 (86.4%)	
Hospital 2	3.0 (13.6%)	3.0 (13.6%)	3.0 (13.6%)	

*CPM, continuous passive motion; CAMuni, continuous active motion unilateral; CAMbi, continuous active motion bilateral; p, probability value; ICD, International Statistical Classification of Diseases and Related Health Problems; A00–B99, Certain infectious and parasitic diseases; C00–D48, Neoplasms; D50–D89, Diseases of the blood and blood-forming organs and certain disorders involving the immune mechanism; E00–E90, Endocrine, nutritional and metabolic diseases; F00–F99, Mental and behavioral disorders; G00–G99, Diseases of the nervous system; H00–H59, Diseases of the eye and adnexa; H60–H95, Diseases of the ear and mastoid process; I00–I99, Diseases of the circulatory system; J00–J99, Diseases of the respiratory system; K00–K93, Diseases of the digestive system; M00–M99, Diseases of the musculoskeletal system and connective tissue; N00–N99, Diseases of the genitourinary system; R00–R99, Symptoms, signs and abnormal clinical and laboratory findings, not elsewhere classified; Z00–Z99, Factors influencing health status and contact with health services*.

† *Denotes a statistical tendency toward a significant difference between groups (p ≤ 0.100). Values are presented as means (standard deviation): analysis of variance*.

‡ *Values are presented as means (standard deviation): analysis of variance*.

¥1 *Fisher's exact test*;

¥2 *Pearson chi-squared test: Values are presented as number (%)*.

§ *Denotes the number (%) of missing data within this data set*.

Table 2 shows the measures of clinical, functional, and quality of life outcomes at pre-test. Tables 3, 4 present the results of the mITT analyses for post- and follow-up tests, respectively. The clinical relevance (Cohen's *d* effect sizes) and statistical significance (*p*-values) for *post-hoc* comparisons between groups are provided in Table 5.

**Table 2:** Measures of clinical, functional, and quality of life outcomes at pre-test (modified intention-to-treat analysis).

	**Pre-test^‡^**		
	**CPM** ** (*n* = 22)**	**CAMuni** ** (*n* = 22)**	**CAMbi** ** (*n* = 22)**
**Range of motion (°)**			
Active knee flexion	110.33 (14.36)	113.77 (15.59)	116.17 (8.55)
Active knee extension	6.41 (6.27)	4.75 (3.69)	5.35 (4.01)
**Swelling (cm)**	46.16 (5.14)	45.90 (4.90)	45.45 (3.98)
**Knee pain (cm)**	4.67 (2.50)	5.07 (2.54)	3.68 (2.30)
**Timed-up-and-go performance (s)**	10.18 (2.46)	8.82 (2.87)	9.38 (2.89)
Missing data^§^	0.0 (0.0%)	1.0 (4.5%)	1.0 (4.5%)
**Stair-climbing performance (s)**	27.13 (12.34)	23.28 (12.92)	22.30 (6.83)
Missing data^§^	2.0 (9.1%)	1.0 (4.5%)	1.0 (4.5%)
**iMVT (N·m)**	110.88 (43.18)	114.05 (50.26)	130.79 (53.41)
**SF-36**			
SF-36 score	45.88 (15.17)	41.79 (14.43)	46.24 (14.28)
Physical health	31.65 (14.65)	29.28 (11.67)	33.43 (13.98)
Mental health	58.96 (19.24)	53.92 (20.23)	58.90 (18.11)
Missing data^§^	0.0 (0.0%)	1.0 (4.5%)	0.0 (0.0%)
**CRP (mg/dl.)**	3.44 (2.99)	5.34 (10.35)	4.52 (4.81)

*CPM, continuous passive motion; CAMuni, continuous active motion unilateral; CAMbi, continuous active motion bilateral; iMVT, isometric maximal voluntary torque; CRP, C-reactive protein*.

‡ *Values are presented as means (standard deviation)*.

§ *Denotes the number (%) of missing data within this data set. Multiple imputation was not possible because data were available for only one or even no measuring point*.

**Table 3:** Measures of clinical, functional, and quality of life outcomes at post-test (modified intention-to-treat analysis).

	**Post-test**									
	**CPM** ** (*n* = 22)**	**CAMuni** ** (*n* = 22)**	**CAMbi** ** (*n* = 22)**	**Mean Difference (95% CI)**			***F***	***p***	***$\eta_{p}^{2}$***	***f***
				**CPM–CAMuni**	**CPM–CAMbi**	**CAMbi–CAMuni**				
**Hospital stay (d)^‡^**	11.50 (2.32)	10.77 (2.27)	10.23 (1.48)	0.73 (−0.80; 2.25)	1.27 (−0.25; 2.80)^++^	−0.55 (−2.07; 0.98)	2.115	0.129	0.063	0.259
**Number of interventions**										
Operated leg^‡^	18.91 (3.22)	17.91 (1.82)	16.27 (2.82)	1.00 (−0.99; 2.99)	2.64 (0.65; 4.62)^+++^	−1.64 (−3.63; 0.35)^++^	5.41	0.007	0.147	0.415
Non-operated leg	n.a.	n.a.	6.50 (1.50)	–	–	–	–	–	–	–
**Range of motion (°)**										
Active knee flexion^¥^	80.04 (12.09)	82.51 (11.77)	89.04 (11.60)	−2.47 (−11.68; 6.76)	−9.00 (−18.03; 0.04)^++^	6.53 (−2.16; 15.21)^++^	3.334	0.043	0.106	0.344
Active knee extension^¥1^	4.86 (3.14)	4.67 (3.11)	3.32 (3.03)	0.20 (−2.22; 2.61)	1.55 (−0.77; 3.87)^++^	−1.35 (−3.64; 0.94)^++^	1.580	0.564	0.024	0.157
**Swelling (cm)^¥^**	49.33 (2.23)	49.37 (2.21)	48.11 (2.12)	−0.04 (−1.76; 1.68)	1.22 (−0.40; 2.84)^++^	−1.26 (−2.86; 0.34)^++^	2.501	0.091	0.081	0.297
**Knee pain (cm)^¥1^**	3.36 (1.69)	1.61 (1.70)	2.56 (1.74)	1.75 (0.50; 3.01)^+++^	0.80 (−0.49; 2.01)	0.95 (−0.35; 2.25)^++^	3.130	0.051	0.099	0.331
**Physical activity**										
Steps^¥1^	2,952 (3,381)	3,989 (3,350)	3,448 (3,263)	−1,036 (−3,629; 1,555)	−496 (−2,994; 2,002)	−540 (−3,004; 1,923)	1.780	0.178	0.059	0.250
Sit-to-stand-transitions^¥^	209.09 (109.05)	297.17 (108.05)	223.33 (105.23)	−88.08 (−171.69; −4.48)^+++^	−14.25 (−94.83; 66.34)	−73.84 (−153.30; 5.63)^++^	3.980	0.024	0.123	0.375
**Timed-up-and-go performance (s)^¥^**	16.88 (5.08)	17.38 (5.01)	14.15 (4.86)	−0.50 (−4.46; 3.46)	2.73 (−1.07; 6.53)^++^	−3.23 (−6.99; 0.53)^++^	2.659	0.079	0.090	0.315
Missing data^§^	0.0 (0.0%)	1.0 (4.5%)	1.0 (4.5%)	–	–	–	–	–	–	–
**Stair-climbing performance (s)^¥1^**	63.58 (20.49)	56.83 (20.08)	49.73 (19.95)	6.75 (−9.38; 22.88)	13.85 (−2.14; 29.84)^++^	−7.10 (−22.46; 8.26)	4.481	0.016	0.147	0.415
Missing data^§^	2.0 (9.1%)	1.0 (4.5%)	1.0 (4.5%)	–	–	–	–	–	–	–
**iMVT (N·m)^¥1^**	37.60 (22.09)	34.13 (22.15)	42.02 (21.81)	3.47 (−13.50; 20.45)	−4.42 (−21.01; 12.19)	7.89 (−8.78; 24.55)	1.250	0.294	0.043	0.212
**EDC/NFB (d)**^**‡**^	3.41 (1.81)	3.00 (1.81)	3.78 (1.81)	0.41 (−0.93; 1.75)	−0.37 (−1.70; 0.97)	0.78 (−0.56; 2.11)	1.009	0.371	0.032	0.182
**CRP (mg/dl.)^¥^**	62.23 (32.17)	35.78 (31.73)	41.09 (30.79)	26.45 (1.70; 51.20)^+++^	21.14 (−2.61; 44.91)^++^	5.31 (−17.96; 28.55)	3.875	0.027	0.122	0.372

*CPM, continuous passive motion; CAMuni, continuous active motion unilateral; CAMbi, continuous active motion bilateral; CI, 95% confidence interval; F, critical F value of the F-distribution (variance of the group means/mean of the within group variances); p, probability value; ηp^2^, effect size partial eta-squared; f, effect size; n. a., not available; iMVT, isometric maximum voluntary torque; EDC, epidural catheter; NFB, nerve femoral block; CRP, C-reactive protein*.

++ *Denotes a medium effect (Cohen's d 0.50–0.79)*.

+++ *Denotes a large effect (Cohen's d ≥ 0.80)*.

‡ *Values are presented as means (standard deviation): analysis of variance*.

¥ *Values are presented as adjusted mean values (adjusted standard deviation) and adjusted mean difference (adjusted 95% CI): analysis of covariance adjusted for pain, swelling, age, BMI, sex, hospital and baseline;*

¥1 *data are not normally distributed and statistical values (F, p, and $\eta_{\text{p}}^{2}$) were calculated from the log-transformed (Lg10) data*.

§ *Denotes the number (%) of missing data within this data set. Multiple imputation was not possible because data were available for only one or even no measuring point*.

**Table 4:** Measures of clinical, functional and quality of life outcomes at follow-up (modified intention-to-treat analysis).

**Follow-up**										
	**CPM** ** (*n* = 22)**	**CAMuni** ** (*n* = 22)**	**CAMbi** ** (*n* = 22)**	**Mean Difference (95% CI)**			***F***	***p***	***$\eta_{p}^{2}$***	***f***
				**CPM–CAMuni**	**CPM–CAMbi**	**CAMbi–CAMuni**				
**Range of motion (°)**										
Active knee flexion^¥^	103.84 (10.18)	106.52 (10.52)	110.14 (10.41)	−3.03 (−10.80; 4.74)	−6.66 (−14.31; 0.99)^++^	3.63 (−4.39; 11.65)	2.297	0.110	0.076	0.287
Active knee extension^¥1^	3.54 (2.82)	2.96 (2.95)	1.76 (2.89)	0.58 (−1.02; 2.17)	1.78 (0.23; 3.33)^++^	−1.20 (−2.86; 0.44)	1.487	0.238	0.068	0.270
**Swelling (cm)^¥^**	45.97 (1.14)	46.36 (1.19)	46.16 (1.17)	−0.39 (−1.26; 0.49)	−0.19 (−1.07; 0.64)	−0.20 (−1.09; 0.74)	0.607	0.549	0.021	0.147
**Knee pain (cm)^¥1^**	1.01 (1.17)	1.59 (1.18)	0.76 (1.19)	−0.58 (−1.45; 0.29)	0.25 (−0.63; 1.12)	−0.83 (1.72; 0.07)^++^	2.039	0.140	0.067	0.268
**Physical activity**										
Steps^¥1^	34,730 (15,273)	38,930 (15,928)	37,050 (15,763)	37,050 (15,763; 7,443)	−2,319 (−13,776; 9,136)	−1,879 (−14,050; 10,291)	0.664	0.519	0.023	0.153
Sit-to-stand-transitions^¥^	338.90 (85.97)	353.84 (92.50)	320.58 (88.77)	−14.94 (−82.98; 53.10)	18.32 (−45.47; 82.11)	−33.26 (−104.20; 7.68)	0.679	0.512	0.024	0.157
**Timed-up-and-go performance (s)^¥^**	9.08 (1.68)	9.30 (1.75)	7.98 (1.71)	−0.22 (−0.53; 2.10)	1.10 (−0.17; 2.37)^++^	−1.32 (−1.68; 1.05)^++^	2.467	0.094	0.084	0.303
Missing data^§^	0.0 (0.0%)	1.0 (4.5%)	1.0 (4.5%)	–	–	–	–	–	–	–
**Stair-climbing performance (s)^¥1^**	23.75 (6.42)	21.82 (6.66)	21.81 (6.58)	1.93 (−3.20; 7.05)	1.94 (−3.09; 6.96)	−0.01 (−5.25; 5.24)	0.729	0.487	0.027	0.167
Missing data^§^	2.0 (9.1%)	1.0 (4.5%)	1.0 (4.5%)	–	–	–	–	–	–	–
**iMVT (N·m)^¥1^**	93.79 (32.69)	101.57 (34.00)	109.13 (34.09)	−7.78 (32.57; 17.02)	−15.34 (−40.23; 9.57)	7.56 (−18.76; 33.86)	1.818	0.172	0.061	0.255
**SF−36^¥^**										
SF-36 score	54.30 (13.27)	67.76 (13.90)	64.81 (13.70)	−13.46 (−23.76; −3.17)^+++^	−10.51 (−20.48; −0.56)^++^	−2.95 (−13.70; 7.81)	6.066	0.004	0.181	0.470
Physical health	44.94 (16.11)	60.58 (16.87)	57.87 (16.63)	−15.64 (−28.13; −3.15)^+++^	−12.93 (−25.03; −0.85)^++^	−2.71 (−15.77; 10.36)	5.783	0.005	0.174	0.459
Mental health	63.44 (15.15)	74.79 (15.87)	73.19 (15.63)	−11.35 (−23.12; 0.41)^++^	−9.75 (−21.12; 1.62)^++^	−1.60 (−13.88; 10.67)	3.538	0.036	0.114	0.359
Missing data^§^	0.0 (0.0%)	1.0 (4.5%)	0.0 (0.0%)	–	–	–	–	–	–	–

*CPM, continuous passive motion; CAMuni, continuous active motion unilateral; CAMbi, continuous active motion bilateral; CI, 95% confidence interval; F, critical F-value of the F-distribution (variance of the group means/mean of the within group variances); p, probability value; ηp^2^, effect size partial eta-squared; f, effect size; iMVT, isometric maximum voluntary torque*.

++ *Denotes a medium effect (Cohen's d 0.50–0.79)*.

+++ *Denotes a large effect (Cohen's d ≥ 0.80)*.

¥ *Values are presented as adjusted mean values (adjusted standard deviation) and adjusted mean difference (adjusted 95% CI): analysis of covariance adjusted for pain, swelling, age, BMI, sex, hospital and baseline*;

¥1 * data are not normally distributed and statistical values (F, p, and $\eta_{\text{p}}^{2}$) were calculated from the log-transformed (Lg10) data*.

§ *Denotes the number (%) of missing data within this data set. Multiple imputation was not possible because data were available for only one or even no measuring point*.

**Table 5:** Clinical relevance (Cohen's *d* effect size) and statistical significance (*p*-value) for *post-hoc* paired comparisons between groups at post-test and follow-up (modified intention-to-treat analysis).

	**Post-test**							**Follow-up**					
	**CPM–CAMuni**		**CPM–CAMbi**		**CAMbi–CAMuni**			**CPM–CAMuni**		**CPM–CAMbi**		**CAMbi–CAMuni**	
	***d***	***p***	***d***	***p***	***d***	***p***		***d***	***p***	***d***	***p***	***d***	***p***
**Hospital stay**	0.32	0.571	0.65^++^	0.128	0.28	0.765							
**Number of interventions**													
**Operated leg**	0.37	0.664	0.99^+++^	0.005^*^	0.61^++^	0.142							
**Range of motion**							**Range of motion**						
Active knee flexion	0.21	0.885	0.76^++^	0.051	0.56^++^	0.194	Active knee flexion	0.26	0.714	0.61^++^	0.105	0.35	0.611
Active knee extension	0.06	0.963	0.50^++^	0.927	0.50^++^	0.639	Active knee extension	0.20	0.320	0.62^++^	0.490	0.41	0.997
**Swelling**	0.02	1.000	0.56^++^	0.194	0.58^++^	0.165	**Swelling**	0.33	0.625	0.16	0.905	0.17	0.370
**Knee pain**	1.03^+++^	0.054	0.47	1.000	0.55^++^	0.306	**Knee pain**	0.49	1.000	0.21	0.441	0.70^++^	0.171
**Physical activity**							**Physical activity**						
Steps	0.31	0.429	0.15	0.206	0.16	0.973	Steps	0.27	0.653	0.15	0.759	0.12	0.998
Sit-to-stand-transitions	0.81^+++^	0.036^†^	0.33	0.963	0.69^++^	0.076	Sit-to-stand-transitions	0.17	0.932	0.21	0.861	0.37	0.584
**Timed-up-and-go performance**	0.10	0.986	0.55^++^	0.227	0.65^++^	0.112	**Timed-up-and-go performance**	0.13	0.376	0.65^++^	0.106	0.76^++^	0.921
**Stair-climbing performance**	0.33	0.472	0.68^++^	0.013^*^	0.35	0.265	**Stair-climbing performance**	0.30	0.640	0.30	0.692	0.00	0.999
**iMVT**	0.16	0.801	0.20	0.317	0.36	0.850	**iMVT**	0.23	0.443	0.46	0.215	0.22	0.970
**EDC/NFB**	0.23	0.838	0.23	0.880	0.43	0.409	**SF-36**						
**CRP**	0.82^+++^	0.033^†^	0.67^++^	0.095	0.17	0.924	SF-36 score	0.99^+++^	0.006^*^	0.78^++^	0.035^†^	0.21	0.877
							Physical health	0.95^+++^	0.010^*^	0.79^++^	0.032^†^	0.16	0.942
							Mental health	0.73^++^	0.061	0.63^++^	1.113	0.10	0.984

*CPM, continuous passive motion; CAMuni, continuous active motion unilateral; CAMbi, continuous active motion bilateral; d, Cohen's d effect size; p, probability value; iMVT, isometric maximal voluntary torque; EDC, epidural catheter; NFB, nerve femoral block; CRP, C-reactive protein*.

++ *Denotes a medium effect (Cohen's d 0.50–0.79)*.

+++ *Denotes a large effect (Cohen's d ≥ 0.80)*.

* *Denotes a significant difference between groups (p ≤ 0.025); alpha-adjustment for conducting two ANCOVA's p ≤ 0.050/2 = 0.025*.

† *Denotes a statistical tendency toward a significant difference between groups (p ≤ 0.050)*.

The CAMbi group revealed less interventions for the operated leg compared to the CPM group (large effect; −14.0%) and CAMuni group (medium effect; −9.2%) because a few patients of the CAMbi group refused to participate in individual interventions due to the high training volume (Table 3).

### Hypothesis I–Active Is Superior to Passive Motion Treatment (CAM Treatment vs. CPM Treatment)

#### Primary Outcome

The results showed clinically relevant differences between CAMbi and CPM treatment in the primary outcome. The data analyses revealed medium effects for a 9.0° (+11.2%) and a 6.3° (+6.1%) greater active knee flexion ROM in the CAMbi compared to the CPM group at post- and follow-up-tests, respectively.

**Figure 3:**
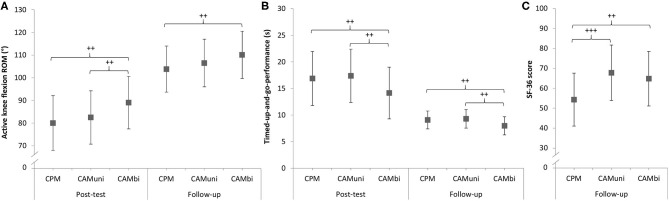
Results of the modified intention-to-treat analyses for **(A)** the primary outcome active knee flexion range of motion (ROM), **(B)** timed-up-up-and-go-performance and **(C)** quality of life (SF-36 score) at post- and follow-up tests, respectively. CPM, continuous passive motion; CAMuni, continuous active motion unilateral; CAMbi, continuous active motion bilateral; + Denotes the clinical relevance (Cohen's *d* effect size) for *post-hoc* comparisons between groups: ++ medium effect (Cohen's *d* 0.50–0.79), +++ large effect (Cohen's *d* ≥ 0.80).

No relevant between-group differences were found for knee flexion ROM between CAMuni vs. CPM treatment at post- and follow-up-tests (Tables 3–5; Figure 3A).

#### Secondary Outcomes

At post-test, large effects were found for reduced knee pain (−52.1%), larger number of sit-to-stand-transitions (+42.1%), and a lower CRP value (−42.5%) in favor of CAMuni treatment as compared to CPM treatment.

At follow-up-test, patients of the CAMuni treatment reported a higher quality of life (SF-36 score; large effect; +24.8%), resulting from a better physical (SF-36 subscale score; large effect; +34.8%) and mental health (SF-36 subscale score; medium effect; +17.9%) following CAMuni as compared to CPM treatment.

At post-test, medium effect sizes were found for stair-climbing performance and timed-up-and-go performance when comparing CAMbi with CPM treatment. The patients of the CAMbi goups needed less time to climb stairs (−21.8%) and to perform the timed-up-and-go test (−16.2%) than participants of the CPM treatment. Moreover, analyses revealed medium effects for a shorter hospital stay (−11.0%), improved knee extension ROM (+31.7%), reduced swelling (−2.5%), and a lower CRP value (−34.0%) in favor of the CAMbi treatment.

At follow-up-test, medium effects were observed for a better timed-up-and-go performance (+12.1%), higher quality of life (SF-36 score; +19.4%), and better physical health (SF-36 subscale score; +28.8%) in the CAMbi treatment compared to the CPM treatment.

The results are presented in Tables 3–5 and Figures 3B,C.

### Hypothesis II–Bilateral Active Motion Treatment Is More Effective Than Unilateral Active Motion Treatment (CAMbi vs. CAMuni Treatment)

#### Primary Outcome

The comparison of CAMbi vs. CAMuni treatment showed a practically relevant higher knee flexion ROM of 6.5° for CAMbi compared to the CAMuni treatment at post-test (medium effect; +7.9%), but not at follow-up-test (Tables 3–5; Figure 3A).

#### Secondary Outcomes

At post-test, medium effect sizes were found for improved active knee extension ROM (+28.9%), reduced swelling (-2.6%) and better timed-up-and-go performance (+18.6%) following CAMbi compared to CAMuni treatment.

In contrast, patients of the CAMbi treatment performed fewer sit-to-stand-transitions than CAMuni group patients in the post-test (medium effect; −24.8%) and follow-up-test (medium effect; −9.4%). Furthermore, patients of the CAMbi treatment reported to have more knee pain (medium effect; +59%) at the post-test. This effect was reversed in the follow-up-test, i.e., patients of the CAMbi treatment had less pain compared to participants of the CAMuni treatment (medium effect; −52.2%).

The results are presented in Tables 3–5 and Figures 3B,C.

### Sensitivity Analysis

There was a consistency between the results of primary analysis (mITT analysis) and sensitivity analysis (PP analysis) for the primary outcome and most secondary outcomes at post- and follow-up-test [see Supplementary File 2 (Tables S4–S7)].

Results of both analyses differed considerably for the comparison of stair-climbing performance between CPM and CAMbi treatment at post-test. The mITT approach showed large treatment effects (*d* = 0.81; *p* = 0.013) compared to the PP analysis (*d* = 0.26; *p* = 1.000).

At follow-up, the mITT approach tended to underestimate the effect for the comparison of Timed-up-and-go performance (PP: *d* = 1.20; *p* = 0.101 vs. mITT: *d* = 0.65; *p* = 0.106) between CPM and CAMbi treatment. A similar trend was observed for the comparison of active knee extension ROM between CPM and CAMbi treatment (PP: *d* = 0.92; *p* = 0.015 vs. mITT: *d* = 0.62; *p* = 0.490) and between CAMbi and CAMuni treatment (PP: *d* = 0.70; *p* = 0.116 vs. mITT: *d* = 0.41; *p* = 0.997). Furthermore, the mITT analysis was not robust to the sensitivity analysis regarding the quality of life outcomes when comparing CPM with CAMbi. The SF-36 score (PP: *d* = 0.59; *p* = 0.263 vs. mITT: *d* = 0.78; *p* = 0.035) and the subscales mental health (PP: *d* = 0.51; *p* = 0.416 vs. mITT: *d* = 0.63; *p* = 1.113) and physical health (PP: *d* = 0.50; *p* = 0.457 vs. mITT: *d* = 0.79; *p* = 0.032) showed larger treatment effects when applying the mITT approach compared to PP.

## Discussion

The present randomized controlled clinical study compared the effectiveness of standard CPM treatment (affected leg 3 ×30 min/day for 8 days) with CAMuni treatment (affected leg 3 ×30 min/day for 8 days), and CAMbi treatment (affected leg 3 ×30 min/day, unaffected leg 1 ×30 min/day for 8 days) in the early post-operative rehabilitation following TKA.

We hypothesized that (I) voluntary muscle activation of the operated leg during the CAM treatments is more effective for restoring function than standard CPM treatment, and (II) positive cross education effects from the unaffected to the affected leg during CAMbi treatment would further promote rehabilitation after TKA.

### Hypothesis I–Active Is Superior to Passive Motion Treatment (CAM vs. CPM Treatment)

#### Primary Outcome

For the primary outcome variable, the first hypothesis can only be confirmed partially. No relevant difference between CAMuni and CPM treatment was observed, while CAMbi treatment was superior to CPM treatment in improving active knee flexion ROM. An increase in knee flexion ROM of more than 5° is required to justify the added time and costs for a motion treatment after TKA (5). Compared to the CPM treatment, the CAMbi treatment resulted in an improved active knee flexion ROM of 9.0° and 6.3° at post- and follow-up test, respectively, indicating that the difference between both treatments is of clinical relevance.

#### Secondary Outcomes

Both CAM treatments were superior to the CPM treatment at post-test. Compared with CAMbi and CPM, the CAMuni group was physically more active (larger number of sit-to-stand-transitions) and reported less knee pain during hospital stay. The inflammatory marker CRP was lower in both CAM groups compared with CPM. Further differences were found for the comparison of CAMbi with CPM. We observed an improved active knee extension ROM, reduced knee joint swelling, a shorter hospital stay, and differences in functional measures (i.e., increased stair-climbing performance and timed-up-and-go performance) in favor of CAMbi as compared to CPM treatment at post-test. Three months after TKA, participants of the CAMbi group showed a better timed-up-and-go performance compared to the CPM group. Furthermore, patients of both CAM groups reported a higher quality of life (SF-36 score).

### Possible Mechanisms Mediating the Positive Effects of CAM Treatments Compared to CPM

#### Preserved Neuromuscular Function of the Trained, Affected Leg

Patients of the CPM group do not actively participate in rehabilitation because the knee joint is passively mobilized. We assumed that low-load resistance training of the affected leg (CAM treatments) may enhance muscle anabolic sensitivity and preserve muscle mass and function during hospital stay (6). However, exercises with a CAM machine, such as the CAMO®ped device, are guided movements, which require a low degree of knee joint stabilization and are therefore less functional. This may be the reason why no further differences in functional outcomes between CAMuni and CPM were observed. The present results are consistent with previous studies in which no relevant effect was found on the primary outcome knee flexion ROM between CAMuni treatment (operated leg) with a comparable device (slider board) and CPM treatment (7, 8). Only one study documented a short-term effect on passive knee flexion ROM after a CAMuni treatment using sling exercises (9). CAMuni exercises with an unstable support (sling) require dynamic joint stabilization, which may be responsible for this positive effect.

#### Exercise-Induced Hypoalgesia

Voluntary muscle activation during the CAM treatments may have stimulated pain modulating processes resulting in an exercise-induced hypoalgesia (39). However, only patients of the CAMuni group reported lower knee pain compared to the CPM patients. There was no difference in knee pain between CAMbi and CPM, suggesting that exercise-induced hypoalgesia is an unlikely mechanism for improved function.

#### Anti-inflammatory Effect of Resistance Training

Peak CRP levels after TKA are usually present at the second and third post-operative day and reduce abruptly if there are no post-operative complications (30). Long-term resistance training may have anti-inflammatory effects (reduced CRP value) in older adults (40), while evidences on short-term responses to CRP occurring hours and days after exercise are inconsistent due to the lack and heterogeneity of studies (41). However, the inflammatory marker CRP was lower in patients of both CAM groups compared to the CPM group at the fifth post-operative day. Thus, the present data suggest that a CAM treatment may reduce inflammation period and support the healing process, which in turn may contribute to increased physical function.

#### Increased Self-Efficacy

It is likely that the CAM treatments have stimulated motivational processes and the ability to overcome stressful situations that may arise from disease and surgery. An increased self-efficacy and mood of patients involved in CAM treatments may have contributed to a better quality of life 3 months after surgery (42). However, further studies are needed to verify this effect. Sensitivity analysis showed that the mITT approach tends to overestimate the difference in quality of life outcomes, especially for the comparison of CPM and CAMbi treatment.

### Hypothesis II–Bilateral Active Motion Treatment Is More Effective Than Unilateral Active Motion Treatment (CAMbi vs. CAMuni Treatment)

#### Primary Outcome

The current study is the first to investigate the effectiveness of a CAMbi treatment in patients after TKA. According to the hypothesis, we observed that CAMbi treatment is more effective than CAMuni treatment in improving active knee flexion ROM. We found a clinically relevant higher flexion ROM of 6.5° in patients of the CAMbi group at post-test.

#### Secondary Outcomes

The results of the secondary outcomes further indicate that the early rehabilitation process following TKA may primarily benefit from the CAMbi intervention. Besides the positive effect of CAMbi treatment on the primary outcome active knee flexion ROM, we observed improved active knee extension ROM, decreased knee joint swelling, and better timed-up-and-go performance compared to CAMuni at post-test. It has been shown that increased swelling is related to functional impairments (43), thus, better timed-up-and-go performance might be partially related to reduced swelling.

However, contrary to the hypothesis, patients of the CAMbi treatment had more knee pain compared to CAMuni group patients at post-test. Three months after TKA, this effect was reversed, i.e., patients of the CAMbi group had less knee pain. The lower long-term knee pain found in the CAMbi group might be of higher relevance for the patients ADL and physical activity behavior.

Furthermore, CAMbi group patients were physically less active (smaller number of sit-to-stand-transitions) during hospitalization compared to patients of the CAMuni group, which might be related to the higher total training volume (see limitations section).

### Possible Mechanisms Mediating the Positive Effects of CAMbi Compared to CAMuni

#### Cross Education Effect

We assumed that patients in the CAMbi group could benefit from transfer effects from the additional training of the unaffected leg to the affected leg (10, 11, 44, 45). Only a few studies analyzed cross education effects in orthopedic patient populations [ACL reconstruction (17–19); distal radius fracture (16); knee osteoarthrosis (20)]. Papandreou et al. investigated the effects of an eccentric cross education intervention in highly trained soldiers after ACL reconstruction 9 weeks after surgery (3 and 5 times/week for 8 weeks; 5 sets of 6 repetitions at 80% of eccentric maximal voluntary contraction) (19). They found improvements (i) in quadriceps reaction time at 90° of knee flexion at a training frequency of 3 times/week and (ii) in the ability to manage everyday life (Lysholm questionnaire) at training frequencies of 3 and 5 times/week. In 2013, the authors further analyzed the effects of the same eccentric cross education intervention after ACL reconstruction on neuromuscular function in soldiers (3 and 5 times/week for 8 weeks; 5 sets of 6 repetitions at 80% of eccentric maximal voluntary contraction) and found (i) a strength-sparing effect and (ii) reduced asymmetry of quadriceps muscle strength between the injured and uninjured leg at both training frequencies (3 and 5 times/week) (18).

Conversely, recent studies by Zult et al. showed that patients (recreational athletes) who participated in a cross education intervention after ACL reconstruction (2 times/week for 12 weeks; 3 sets of 8 to 12 concentric/eccentric contractions) experienced (i) increased limb asymmetry (9–10%) 5 and 12 weeks after surgery and (ii) reduced voluntary activation of the knee extensors of the reconstructed leg (−6%) 12 weeks after ACL reconstruction compared with the control group (17, 46). Furthermore, the cross education intervention did not accelerate recovery of neuromuscular function (i.e., maximal quadriceps strength, force control, proprioception, and dynamic balance).

Twelve weeks after distal radius fracture, Magnus et al. found improved handgrip strength (47%) and wrist flexion/extension ROM (25%) in women older than 50 years after a cross education intervention (5 times/week for 26 weeks; 2 to 5 sets of 8 isometric contractions). However, the authors found no differences in strength and ROM at 9 and 26 weeks after surgery (16). A recent study by Onigbinde et al. analyzed the effect of a unilateral strength training of the unaffected leg in knee osteoarthrosis patients (3 times/week for 6 weeks; 3 sets of 12 isometric maximal voluntary contractions) and found an increase in quadriceps muscle strength of around 20% in both legs indicating a cross education effect (20). Similarly, Harput et al. have found that concentric and eccentric cross education interventions (3 times/week for 8 weeks, 3 sets of 12 isokinetic maximal voluntary contractions) improved isometric maximal voluntary contraction strength compared to standard care after ACL reconstruction (47).

Taken together, the mentioned studies are heterogeneous in terms of patient population (i.e., age, orthopedic disease/injury) and the study design (e.g., methods, outcomes, duration, and intensity of the cross education intervention) and are therefore only partially comparable to our findings for TKA patients.

Adaptations to cross education include functional and structural changes within the neuromuscular system (10, 11). First, modulations along the neuroaxis (i.e., increased corticospinal excitability, reduced cortical inhibition, reduced interhemispheric inhibition, changes in voluntary activation, and new regions of cortical activation) primary contribute to changes within the central nervous system including cortical motor and non-motor regions (10).

Second, cross education can prevent muscle atrophy in the untrained leg, which might be mediated by an altered balance between muscle protein synthesis and breakdown (48, 49).

However, the modulation of sensory feedback due to knee joint swelling, pain, inflammation, joint laxity, damage to joint afferents, and removal of joint structures as a result of knee osteoarthrosis and TKA (1) may have induced changes in the central nervous system and reduced the responsiveness to a cross education intervention. As proposed for patients after ACL reconstruction, modulations in somatosensory and motor areas may have been also modulated in TKA patients that in turn may have reduced the sensitivity for sensory and motor stimuli (17). Altered afferent feedback and changes in sensorimotor area could be an explanation why the cross education intervention did not increase iMVT of the affected leg in the present study. This finding contradicts our assumption and the results of a recent meta-analysis of 96 studies by Green and Gabriel (50). The authors reported a cross education strength gain of 15% in older adults and a 29% increase in patients (50).

#### Preserved Neuromuscular Function of the Trained, Unaffected Leg

Cross education usually occurs with the presence of a training effect in the trained leg. However, episodic muscle disuse as a result of reduced physical activity after surgery may have induced a higher strength loss in the untrained, unaffected leg of the CAMuni group compared to the trained, unaffected leg of the CAMbi group. Physiological consequences of reduced physical activity (i.e., step reduction) contribute to reductions in muscle mass and strength, impaired insulin sensitivity, and an increase in systematic inflammation [for a review see Oikawaet al. (51)]. Thus, preserved muscle function of the trained, unaffected leg in the CAMbi group may have contributed to higher functional outcomes compared to CAMuni.

### Limitations

#### Training Frequency and Duration (Time-Course of Adaptation)

CAMbi group patients received an additional daily intervention for the unaffected leg and may have been more fatigued than CAMuni group patients. Some patients of the CAMbi group actually refused to participate in individual interventions due to the high training volume, resulting in a lower number of interventions for the operated leg compared to the CPM group (−14.0%) and CAMuni group (−9.2%). The higher training volume may also be related to the lower physical activity level (smaller number of sit-to-stand-transitions) of the CAMbi group patients compared to CAMuni during hospitalization.

In the present study, the average number of cross education interventions (training sessions of the unaffected leg) was 6.5. It remains to be discussed if this number is sufficient to induce cross education effects. A recently published study by Brass et el. analyzed the time-course of handgrip force after a ‘traditional' cross education protocol (3 times/week for 6 weeks; 5 sets of 5 isometric maximal voluntary contractions) and a daily cross education treatment (7 times/week for 18 days; 5 sets of 5 isometric maximal voluntary contractions) (52). Significant strength gains in the untrained arm were found after 12 training sessions (i.e., after 45 days) when using the “traditional” protocol (12.5%) and after 15 training sessions (i.e., after 21 days) when using daily intervention (7.8%). When using the daily protocol, the same strength gains have been achieved in half of the duration of the “traditional” cross education protocol. The authors concluded that recovery of strength may be optimized by reducing the rest days between cross education interventions. Thus, the application of daily cross education intervention in TKA patients seems to be optimal for inducing cross education effects. However, the average number of daily training sessions seems to have been too small as a minimum number of at least 15 sessions is required to achieve improvements in strength (52). It becomes apparent that further studies are needed to replicate and extent these findings.

#### Training Intensity

The CAM device has only four predefined levels of difficulty. To determine the training intensity, we analyzed muscle activity of the affected leg during the CAM treatment and found a %AEMG_iMVT_ of around 33% in the vastus lateralis muscle during knee extensions. Thus, the CAM treatment of the affected leg can be defined as low-load resistance training (6). However, to increase efficiency of the CAM treatments, future studies should use CAM devices that allow for a progressive increase of training load and the training intensity should be determined depending on pre-operative iMVT.

#### Supervisor During CAM Treatments

In order to ensure that CAM treatments were carried out correctly, permanent presence of a supervisor had to be guaranteed. It could be that the CAM patients were positively influenced (motivation) by the presence of an instructor. Due to the limited personnel capacities and the independent applicability of the CPM machine, it was not possible to supervise the CPM treatment. Further research is necessary to control for such supervisor effects, for example by investigating the effects of low-load resistance training with autonomic CAM devices or by supervising CPM treatment in the same way as necessary for CAM treatment.

#### Potential Effects of Subjects' Trait and State Properties on Performance Measures

It has been shown that trait and state characteristics of subjects are related to performance measures. For example, state fatigue can modulate endurance and dynamic balance performance (53, 54). Furthermore, it has been recently shown that trait self-control is a predictor of endurance performance in patients with multiple sclerosis (55). The influence of trait and state properties on performance measures in TKA patients is unknown and should be considered in future studies.

#### Limitations of the mITT Approach

We decided to conduct a mITT analysis, meaning that 19 patients who were randomized but never received any treatment were excluded from the analyses. These modifications were applied to the data *post-hoc*. The reasons for post-randomization exclusion of patients were not related to the treatment. These exclusions can thus be justified as unlikely to bias the results.

## Conclusion

Our findings support the implementation of unilateral (CAMuni) and bilateral alternating (CAMbi) low-load resistance training for restoring function in early rehabilitation programs after TKA. The positive effects of both CAM interventions compared to CPM may be due to (i) preserved neuromuscular function of the trained, affected leg, (ii) the anti-inflammatory effect of resistance training, and/or (iii) increased self-efficacy.

Furthermore, the CAMbi treatment proved to be more effective for recovery than CAMuni. Possible mechanisms mediating the positive effects of CAMbi compared to CAMuni include (i) positive transfer effects from the unaffected to the affected limb (cross education effect) and/or (ii) preserved neuromuscular function of the trained, unaffected leg.

Taken together, aside from knee osteoarthrosis and TKA itself, reduced physical activity during early rehabilitation leads to structural and functional changes within the nervous, muscle, and endocrine systems. Since the greatest loss of physical function occurs in the first month following TKA, low-load resistance training of the affected leg (CAMuni) and especially of both legs (CAMbi) seems to be a promising and viable therapeutic approach to restore and preserve function during early rehabilitation after TKA.

## Data Availability Statement

The raw data supporting the conclusions of this article will be made available by the authors, without undue reservation.

## Ethics Statement

The studies involving human participants were reviewed and approved by Ethics Committee of the University Medicine Rostock. The patients/participants provided their written informed consent to participate in this study.

## Author Contributions

RJ: conceptualization, methodology, software, formal analysis, investigation, data curation, original draft preparation, and review and editing. TS: conceptualization, methodology, formal analysis, investigation, data curation, and review and editing. MB: conceptualization, methodology, formal analysis, investigation, data curation, review and editing, and funding acquisition. FF: methodology, software, formal analysis, and review and editing. PB: methodology and review and editing. RB: methodology, software, funding acquisition, and review and editing. WM and RS: methodology, funding acquisition, and review and editing. AM-M: conceptualization, methodology, software, formal analysis, investigation, data curation, original draft preparation, funding acquisition, and review and editing. All authors contributed to the article and approved the submitted version.

## Conflict of Interest

The authors declare that the research was conducted in the absence of any commercial or financial relationships that could be construed as a potential conflict of interest.

## Abbreviations

ACL: anterior cruciate ligament
ADL: activities of daily living
AEMG: averaged electromyographic signal
ANCOVA: analysis of covariance
BMI: body mass index
CAMbi: continuous active motion bilateral alternating of the affected and unaffected leg
CAMuni: continuous active motion unilateral of the affected leg
CONSORT: Consolidated Standards of Reporting Trials
CPM: continuous passive motion
CRP: C-reactive protein
CV: coefficient of variation
EDC: epidural catheter
ICC: intraclass correlation coefficient
ICD: International Statistical Classification of Diseases and Related Health Problems
iMVT: isometric maximal voluntary torque
mITT: modified intention-to-treat analysis
LLRT: low-load resistance training
PP: per-protocol analysis
Qol: quality of life
ROM: range of motion
ROM_Ext_: maximal active knee extension ROM
ROM_Flex_: maximal active knee flexion ROM
TKA: total knee arthroplasty.
