# Supplementary material for: Low-Load Unilateral and Bilateral Resistance Training to Restore Lower Limb Function in the Early Rehabilitation After Total Knee Arthroplasty: A Randomized Active-Controlled Clinical Trial
Source: Front Med (Lausanne). 2021 Jun 22;8:628021. doi: 10.3389/fmed.2021.628021 (PMC8257942; doi:10.3389/fmed.2021.628021)
Supplement: Supplementary file 1 [file Data_Sheet_1.docx]

**Low-load unilateral and bilateral resistance training to restore lower limb function in the early rehabilitation after total knee arthroplasty: a randomized active-controlled clinical trial**

**Running head:** Low-load resistance training and rehabilitation after TKA

**Supplementary Data Sheet 1: Standardized in-hospital physiotherapy**

__________________________________________________________________

# Standardized in-hospital physiotherapy

Supplementary Table S1. Standardized in-hospital physiotherapy on the first postoperative day.

| service | explanation | time (min) | | treatment goal | locality | therapists | tool |
| --- | --- | --- | --- | --- | --- | --- | --- |
| physiotherapeutic diagnosis | - pain and functional diagnosis | 5 | | - testing of sensibility and motor skills | patient room | 1 |  |
| PT modified according to neurophysiologic principles | - diagnosis based on physiotherapeutic techniques | 15 - 20 | | - blood circulation stimulation - increasing resorption - muscle strengthening | patient room | 1 |  |
| GT (assumption: quadriceps strength non operated leg according to Janda level 4/5) | - sit-to-stand-transition - gait retraining | 10 | | - cardiovascular training - thrombosis prophylaxis - improving self-sufficiency | patient room | 1 - 2 | walking carriage with forearm support,  forearm crutches |
| range of motion documentation | - verification of extension and flexion |  | | - verification of extension and flexion | patient room | 1 | patient record |
| total time (min) |  | 25 - 30 |  | |  |  |  |

Abbreviations: PT, physiotherapy; GT, gait training.

Supplementary Table S2. Standardized in-hospital physiotherapy on the second postoperative day.

| service | explanation | time (min) | | treatment goal | | locality | therapists | tool |
| --- | --- | --- | --- | --- | --- | --- | --- | --- |
| PT modified according to neurophysiologic principles and activities of daily living | - diagnosis based on physiotherapeutic techniques | 15 - 20 | | - thrombosis prophylaxis - improving range of motion - reduction of muscular imbalance - improving self-sufficiency - reduce swelling - if required, strengthening of the upper extremity and the trunk muscles | | patient room | 1 |  |
| GT | - sit-to-stand-transition - GT with tools | 10 | | - cardiovascular training - thrombosis prophylaxis - improving adjustment of gait - improving self-sufficiency | | patient room,  corridor | 1 | walking carriage with forearm support,  rollator,  forearm crutches |
| range of motion documentation | - verification of extension and flexion |  | | - verification of extension and flexion | | patient room | 1 | patient record |
| total time (min) |  | 25 - 30 |  | |  | |  |  |

Abbreviations: PT, physiotherapy; GT, gait training.

Supplementary Table S3. Standardized in-hospital physiotherapy from the third postoperative day until two days before discharge.

| service | explanation | time (min) | treatment goal | | locality | therapists | | tool | |
| --- | --- | --- | --- | --- | --- | --- | --- | --- | --- |
| PT modified according to neurophysiologic principles | - diagnosis based on physiotherapeutic techniques | 15 - 20 | - thrombosis prophylaxis - improving range of motion - reduction of muscular imbalance - improving self-sufficiency - reduce swelling - if required, strengthening of the upper extremity and the trunk muscles | | patient room or treatment room | 1 | |  | |
| GT | - GT with tools und stairs | 10 | - cardiovascular training - thrombosis prophylaxis - improving adjustment of gait - improving self-sufficiency on the stairs | | corridor,  stair | 1 | | walking carriage with forearm support,  forearm crutches | |
| range of motion documentation | - verification of extension and flexion |  | - verification of extension and flexion | | patient room | 1 | | patient record | |
| total time (min) |  | 25 - 30 |  |  | | |  | |  |

Abbreviations: PT, physiotherapy; GT, gait training.
