# Supplementary material for: Low-Load Unilateral and Bilateral Resistance Training to Restore Lower Limb Function in the Early Rehabilitation After Total Knee Arthroplasty: A Randomized Active-Controlled Clinical Trial
Source: Front Med (Lausanne). 2021 Jun 22;8:628021. doi: 10.3389/fmed.2021.628021 (PMC8257942; doi:10.3389/fmed.2021.628021)
Supplement: Supplementary file 2 [file Data_Sheet_2.docx]

**Low-load unilateral and bilateral resistance training to restore lower limb function in the early rehabilitation after total knee arthroplasty: a randomized active-controlled clinical trial**

**Running head:** Low-load resistance training and rehabilitation after TKA

**Supplementary Data Sheet 2: Results of per-protocol analysis**

___________________________________________________________________

# Results of per-protocol analyses

Supplementary Table S4. Measures of clinical, functional and quality of life outcomes at pre-test (per-protocol analysis).

| **Pre-test** ^‡^ | | | | |
| --- | --- | --- | --- | --- |
|  | **CPM**  (n = 20) | **CAMuni**  (n = 20) | **CAMbi**  (n =20) |  |
|  |  |  |  |  |
| Range of motion (°) |  |  |  |  |
| Active knee flexion | 110.33 (14.36) | 113.77 (15.59) | 116.17 (8.55) |  |
| Active knee extension | 6.41 (6.27) | 4.75 (3.69) | 5.35 (4.01) |  |
| Swelling (cm) | 46.16 (5.14) | 45.90 (4.90) | 45.45 (3.98) |  |
| Knee pain (cm) | 4.67 (2.50) | 5.07 (2.54) | 3.68 (2.30) |  |
| Timed-up-and-go performance (s) | 10.18 (2.46) | 8.82 (2.95) | 9.38 (2.89) |  |
| Missing data ^§^ | 0.0 (0.0%) | 2.0 (9.1%) | 1.0 (4.5%) |  |
| Stair-climbing performance (s) | 26.18 (12.72) | 22.98 (13.18) | 22.08 (6.93) |  |
| Missing data ^§^ | 5.0 (22.7%) | 2.0 (9.1%) | 2.0 (9.1%) |  |
| iMVT (N∙m) | 110.88 (43.18) | 114.05 (50.26) | 130.79 (53.41) |  |
| SF-36 |  |  |  |  |
| SF-36 score | 45.69 (15.52) | 41.48 (14.74) | 46.49 (14.81) |  |
| Physical health | 31.80 (15.00) | 29.61 (11.88) | 33.78 (14.49) |  |
| Mental health | 59.58 (19.49) | 53.35 (20.58) | 59.20 (19.01) |  |
| Missing data ^§^ | 1.0 (4.5%) | 2.0 (9.1%) | 2.0 (9.1%) |  |
| CRP (mg/dl.) | 3.44 (2.92) | 5.34 (10.11) | 4.52 (4.70) |  |
|  |  |  |  |  |

Abbreviations: CPM, continuous passive motion; CAMuni, continuous active motion unilateral; CAMbi, continuous active motion bilateral; iMVT, isometric maximal voluntary torque; CRP, C-reactive protein.

^‡^ Values are presented as means (standard deviation).

^§^ Denotes the number (%) of missing data within this data set.

Supplementary Table S5. Measures of clinical, functional and quality of life outcomes at post-test (per-protocol analysis).

| **Post-test** | | | | | | | | | | |
| --- | --- | --- | --- | --- | --- | --- | --- | --- | --- | --- |
|  | **CPM**  (n = 20) | **CAMuni**  (n = 20) | **CAMbi**  (n = 20) | **Mean Difference (95% CI)** | | | **F** | ***p*** | ***η_p_^2^*** | ***f*** |
|  |  |  |  | **CPM - CAMuni** | **CPM - CAMbi** | **CAMbi - CAMuni** |  |  |  |  |
|  |  |  |  |  |  |  |  |  |  |  |
| Hospital stay (d) ^‡^ | 11.50 (2.32) | 10.77 (2.27) | 10.23 (1.48) | 0.73 (-0.80; 2.26) | 1.27 (-0.26; 2.80)^++^ | -0.54 (-2.07; 0.98) | 2.115 | 0.129 | 0.063 | 0.259 |
| Range of motion (°) |  |  |  |  |  |  |  |  |  |  |
| Active knee flexion ^¥^ | 80.04 (12.09) | 82.51 (11.77) | 89.04 (11.60) | -2.47 (-11.68; 6.76) | -9.00 (-18.03; 0.04)^++^ | 6.53 (-2.16; 15.21)^++^ | 3.334 | 0.043 | 0.106 | 0.344 |
| Active knee extension ^¥1^ | 4.86 (3.14) | 4.67 (3.11) | 3.32 (3.03) | 0.19 (-2.22; 2.61) | 1.54 (-0.77; 3.87)^++^ | -1.35 (-3.64; 0.94)^++^ | 0.580 | 0.564 | 0.024 | 0.157 |
| Swelling (cm) ^¥^ | 49.33 (2.23) | 49.37 (2.21) | 48.11 (2.12) | -0.04 (-1.76; 1.68) | 1.22 (-0.40; 2.84)^++^ | -1.26 (-2.86; 0.34)^++^ | 2.501 | 0.091 | 0.081 | 0.297 |
| Knee pain (cm) ^¥1^ | 3.36 (1.69) | 1.61 (1.70) | 2.56 (1.74) | 1.75 (0.50; 3.01)^+++^ | 0.80 (-0.49; 2.01) | 0.95 (-0.35; 2.25)^++^ | 3.130 | 0.051 | 0.099 | 0.332 |
| Physical activity |  |  |  |  |  |  |  |  |  |  |
| Steps ^¥1^ | 2964 (3407) | 3998 (3374) | 3413 (3283) | -1034(-3656; 1587) | -449 (-3000; 2132) | -585 (-3100; 1998) | 1.600 | 0.211 | 0.054 | 0.239 |
| Sit-to-stand-transitions ^¥1^ | 208.69 (109.94) | 296.83 (108.89) | 223.89 (105.95) | -88.14 (-172.73; -3.54)^+++^ | -15.20 (-97.52; 67.14) | -72.94 (-154.07; 8.18)^++^ | 3.255 | 0.046 | 0.104 | 0.341 |
| Missing data ^§^ | 0.0 (0.0%) | 0.0 (0.0%) | 1.0 (4.5%) | - | - | - | - | - | - | - |
| Timed-up-and-go performance (s) ^¥1^ | 16.78 (5.45) | 17.54 (5.33) | 14.15 (5.14) | -0.76 (-5.30; 3.77) | 2.63 (-1.79; 7.05)^++^ | -3.39 (-7.63; 0.84)^++^ | 1.838 | 0.170 | 0.073 | 0.281 |
| Missing data ^§^ | 3.0 (13.6%) | 1.0 (4.5%) | 4.0 (18.2%) | - | - | - | - | - | - | - |
| Stair-climbing performance (s) ^¥1^ | 55.19 (21.52) | 55.16 (20.98) | 49.72 (21.12) | 0.03 (-20.26; 20.33) | 5.47 (-15.24; 26.18) | -5.44 (-23.56; 12.69) | 0.661 | 0.523 | 0.034 | 0.188 |
| Missing data ^§^ | 8.0 (36.4%) | 3.0 (13.6%) | 4.0 (18.2%) | - | - | - | - | - | - | - |
| iMVT (N∙m) ^¥1^ | 37.52 (22.36) | 34.40 (22.34) | 42.15 (21.07) | 3.12 (-14.27; 20.50) | -4.63 (-21.70; 12.45) | 7.75 (-9.49; 24.97) | 1.189 | 0.312 | 0.042 | 0.209 |
| Missing data ^§^ | 0.0 (0.0%) | 1.0 (4.5%) | 1.0 (4.5%) | - | - | - | - | - | - | - |
| EDC/NFB (d) ^‡^ | 3.41 (1.81) | 3.00 (1.81) | 3.78 (1.81) | 0.41 (-0.93; 1.75) | -0.37 (-1.70; 0.97) | 0.78 (-0.56; 2.11) | 1.009 | 0.371 | 0.032 | 0.182 |
| CRP (mg/dl.) ^¥^ | 62.23 (32.17) | 35.78 (31.73) | 41.09 (30.79) | 26.45 (1.70; 51.20)^+++^ | 21.14 (-2.61; 44.91)^++^ | 5.31 (-17.96; 28.55) | 3.875 | 0.027 | 0.122 | 0.372 |
|  |  |  |  |  |  |  |  |  |  |  |

Abbreviations: CPM, continuous passive motion; CAMuni, continuous active motion unilateral; CAMbi, continuous active motion bilateral; CI, 95% confidence interval; F, critical F value of the F-distribution (variance of the group means / mean of the within group variances); *p*, probability value; *ηp^2^*, effect size partial eta-squared; *f*, effect size; iMVT, isometric maximal voluntary torque; EDC, epidural catheter; NFB, nerve femoral block; CRP, C-reactive protein.

^++^ Denotes a medium effect (Cohen’s *d* 0.50 to 0.79).

^+++^ Denotes a large effect (Cohen’s *d* ≥ 0.80).

^‡^ Values are presented as means (standard deviation): analysis of variance.

^¥^ Values are presented as adjusted mean values (adjusted standard deviation) and adjusted mean difference (adjusted 95% CI): analysis of covariance adjusted for pain, swelling, age, BMI, sex, hospital and baseline; ^¥1^ data are not normally distributed and statistical values (F*, p* and *η_p_^2^*) were calculated from the log-transformed (Lg10) data.

^§^  Denotes the number (%) of missing data within this data set.

Supplementary Table S6. Measures of clinical, functional and quality of life outcomes at follow-up (per-protocol analysis).

| **Follow-up** | | | | | | | | | | |
| --- | --- | --- | --- | --- | --- | --- | --- | --- | --- | --- |
|  | **CPM**  **(n = 20)** | **CAMuni**  **(n = 20)** | **CAMbi**  **(n = 20)** | **Mean Difference (95% CI)** | | | **F** | ***p*** | ***η_p_^2^*** | ***f*** |
|  |  |  |  | **CPM - CAMuni** | **CPM - CAMbi** | **CAMbi - CAMuni** |  |  |  |  |
|  |  |  |  |  |  |  |  |  |  |  |
| Range of motion (°) |  |  |  |  |  |  |  |  |  |  |
| Active knee flexion ^¥^ | 102.60 (10.31) | 105.91 (10.80) | 109.55 (10.67) | -3.31 (-11.67; 5.03) | -6.95 (-15.16; 1.24)^++^ | 3.64 (-5.12; 12.39) | 2.212 | 0.120 | 0.081 | 0.297 |
| Active knee extension ^¥1^ | 3.58 (2.12) | 3.14 (2.25) | 1.58 (2.21) | 0.44 (-1.29; 2.16) | 2.00 (0.32; 3.69)^+++^ | -1.56 (-3.40; 0.26)^++^ | 4.573 | 0.015 | 0.155 | 0.428 |
| Missing data ^§^ | 2.0 (9.1%) | 2.0 (9.1%) | 2.0 (9.1%) | - | - | - | - | - | - | - |
| Swelling (cm) ^¥^ | 45.49 (1.19) | 45.82 (1.25) | 45.62 (1.24) | -0.33 (-1.29; 0.63) | -0.13 (-1.08; 0.82) | -0.20 (-1.21; 0.81) | 0.364 | 0.697 | 0.014 | 0.119 |
| Missing data ^§^ | 2.0 (9.1%) | 2.0 (9.1%) | 2.0 (9.1%) | - | - | - | - | - | - | - |
| Knee pain (cm) ^¥1^ | 0.98 (1.22) | 1.56 (1.24) | 0.67 (1.24) | -0.58 (-1.54; 0.38) | 0.31 (-0.65; 1.28) | -0.89 (-1.89; 0.10)^++^ | 2.083 | 0.135 | 0.076 | 0.287 |
| Missing data ^§^ | 2.0 (9.1%) | 2.0 (9.1%) | 2.0 (9.1%) | - | - | - | - | - | - | - |
| Physical activity |  |  |  |  |  |  |  |  |  |  |
| Steps ^¥^ | 34085 (16176) | 39902 (16942) | 37172 (16716) | -5817 (-15843; 7444) | -3087 (-13776; 9137) | -2730 (-14051; 10291) | 0.569 | 0.570 | 0.024 | 0.157 |
| Sit-to-stand-transitions ^¥1^ | 338.90 (85.97) | 351.87 (92.50) | 320.48 (88.77) | -12.97 (-92.53; 66.60) | 18.42 (-55.67; 92.53) | -31.39 (-114.79; 52.01) | 0.368 | 0.694 | 0.016 | 0.128 |
| Missing data ^§^ | 2.0 (9.1%) | 5.0 (22.7%) | 2.0 (9.1%) | - | - | - | - | - | - | - |
| Timed-up-and-go performance (s) ^¥1^ | 9.13 (1.76) | 8.25 (1.85) | 7.00 (1.80) | 0.88 (-0.59; 2.37) | 2.13 (-2.80; 2.55)^+++^ | -1.25 (-1.76; 1.27)^++^ | 2.508 | 0.092 | 0.095 | 0.324 |
| Missing data ^§^ | 3.0 (13.6%) | 2.0 (9.1%) | 2.0 (9.1%) | - | - | - | - | - | - | - |
| Stair-climbing performance (s) ^¥^ | 23.47 (6.51) | 20.52 (6.83) | 21.34 (6.71) | 2.95 (-3.14; 9.05) | 2.13 (-3.84; 8.12) | 0.82 (-5.02; 6.66) | 0.777 | 0.467 | 0.037 | 0.196 |
| Missing data ^§^ | 6.0 (27.3%) | 2.0 (9.1%) | 4.0 (18.2%) | - | - | - | - | - | - | - |
| iMVT (N∙m) ^¥1^ | 91.62 (26.70) | 99.62 (34.00) | 107.26 (34.91) | -8.00 (-34.75; 18.75) | -15.64 (-42.16; 10.88)^++^ | 7.64 (-20.89; 36.17) | 1.076 | 0.349 | 0.041 | 0.207 |
| Missing data ^§^ | 2.0 (9.1%) | 2.0 (9.1%) | 2.0 (9.1%) | - | - | - | - | - | - | - |
| SF-36 ^¥^ |  |  |  |  |  |  |  |  |  |  |
| SF-36 score | 55.38 (14.13) | 67.75 (14.52) | 63.93 (14.65) | -12.37 (-24.12; -0.62)^+++^ | -8.55 (-20.75; 3.64)^++^ | -3.82 (-16.35; 8.72) | 3.632 | 0.035 | 0.142 | 0.407 |
| Physical health | 46.63 (17.35) | 61.38 (17.83) | 55.41 (18.02) | -14.75 (-29.16; -0.34)^+++^ | -8.78 (-23.79; 6.23)^++^ | -5.97 (-21.38; 9.45) | 3.303 | 0.046 | 0.131 | 0.388 |
| Mental health | 64.25 (15.85) | 73.92 (16.25) | 72.54 (16.39) | -9.67 (-22.85; 3.51)^++^ | -8.29 (-21.97; 5.39)^++^ | -1.38 (-15.39; 12.64) | 1.956 | 0.154 | 0.082 | 0.299 |
| Missing data ^§^ | 3.0 (13.6%) | 2.0 (9.1%) | 3.0 (13.6%) | - | - | - | - | - | - | - |
|  |  |  |  |  |  |  |  |  |  |  |

Abbreviations: CPM, continuous passive motion; CAMuni, continuous active motion unilateral; CAMbi, continuous active motion bilateral; CI, 95% confidence interval; F, critical F value of the F-distribution (variance of the group means / mean of the within group variances); *p*, probability value, *ηp^2^*, effect size partial eta-squared; *f*, effect size; iMVT, isometric maximal voluntary torque.

^++^ Denotes a medium effect (Cohen’s *d* 0.50 to 0.79).

^+++^ Denotes a large effect (Cohen’s *d* ≥ 0.80).

^‡^ Values are presented as means (standard deviation): analysis of variance.

^¥^ Values are presented as adjusted mean values (adjusted standard deviation) and adjusted mean difference (adjusted 95% CI): analysis of covariance adjusted for pain, swelling, age, BMI, sex, hospital and baseline; ^¥1^ data are not normally distributed and statistical values (F*, p* and *η_p_^2^*) were calculated from the log-transformed (Lg10) data.

^§^  Denotes the number (%) of missing data within this data set. **Supplementary Table S7.** Clinical relevance (Cohen’s *d* effect size) and statistical significance (*p*-value) for post-hoc paired comparisons between groups at post-test and follow-up (per-protocol analysis).

|  | **Post-test** | | | | | |  | **Follow-up** | | | | | |
| --- | --- | --- | --- | --- | --- | --- | --- | --- | --- | --- | --- | --- | --- |
|  | **CPM - CAMuni** | | **CPM - CAMbi** | | **CAMbi - CAMuni** | |  | **CPM - CAMuni** | | **CPM - CAMbi** | | **CAMbi - CAMuni** | |
|  | ***d*** | ***p*** | ***d*** | ***p*** | ***d*** | ***p*** |  | ***d*** | ***p*** | ***d*** | ***p*** | ***d*** | ***p*** |
|  |  |  |  |  |  |  |  |  |  |  |  |  |  |
| Hospital stay | 0.32 | 0.571 | 0.65^++^ | 0.128 | 0.28 | 0.765 |  |  |  |  |  |  |  |
| Range of motion |  |  |  |  |  |  | Range of motion |  |  |  |  |  |  |
| Active knee flexion | 0.21 | 0.885 | 0.76^++^ | 0.051 | 0.56^++^ | 0.194 | Active knee flexion | 0.31 | 0.989 | 0.66^++^ | 0.122 | 0.34 | 0.925 |
| Active knee extension | 0.06 | 0.963 | 0.50^++^ | 0.927 | 0.50^++^ | 0.639 | Active knee extension | 0.20 | 1.000 | 0.92^+++^ | 0.015* | 0.70^++^ | 0.116 |
| Swelling | 0.02 | 1.000 | 0.56^++^ | 0.194 | 0.58^++^ | 0.165 | Swelling | 0.27 | 1.000 | 0.11 | 1.000 | 0.16 | 1.000 |
| Knee pain | 1.03^+++^ | 0.054 | 0.47 | 1.000 | 0.55^++^ | 0.306 | Knee pain | 0.47 | 1.000 | 0.25 | 0.661 | 0.72^++^ | 0.143 |
| Physical activity |  |  |  |  |  |  | Physical activity |  |  |  |  |  |  |
| Steps | 0.31 | 0.523 | 0.13 | 0.286 | 0.18 | 1.000 | Steps | 0.35 | 0.892 | 0.19 | 1.000 | 0.16 | 1.000 |
| Sit-to-stand-transitions | 0.81^+++^ | 0.052 | 0.14 | 1.000 | 0.68^++^ | 0.212 | Sit-to-stand transitions | 0.15 | 1.000 | 0.21 | 1.000 | 0.35 | 1.000 |
| Timed-up-and-go performance | 0.10 | 1.000 | 0.50^++^ | 0.360 | 0.65^++^ | 0.304 | Timed-up-and-go performance | 0.49 | 0.477 | 1.20^+++^ | 0.101 | 0.69^++^ | 1.000 |
| Stair-climbing performance | 0.44 | 1.000 | 0.26 | 1.000 | 0.26 | 0.979 | Stair-climbing performance | 0.44 | 0.698 | 0.32 | 1.000 | 0.12 | 1.000 |
| iMVT | 0.16 | 1.000 | 0.21 | 0.388 | 0.36 | 1.000 | iMVT | 0.26 | 1.000 | 0.50^++^ | 0.451 | 0.22 | 1.000 |
| EDC/NFB | 0.23 | 0.838 | 0.23 | 0.880 | 0.43 | 0.409 | SF-36 |  |  |  |  |  |  |
| CRP | 0.82^+++^ | 0.033^†^ | 0.67^++^ | 0.095 | 0.17 | 0.924 | SF-36 score | 0.86^+++^ | 0.036^†^ | 0.59^++^ | 0.263 | 0.26 | 1.000 |
|  |  |  |  |  |  |  | Physical health | 0.84^+++^ | 0.043^†^ | 0.50^++^ | 0.457 | 0.33 | 1.000 |
|  |  |  |  |  |  |  | Mental health | 0.60^++^ | 0.224 | 0.51^++^ | 0.416 | 0.01 | 1.000 |
|  |  |  |  |  |  |  |  |  |  |  |  |  |  |

Abbreviations: CPM, continuous passive motion; CAMuni, continuous active motion unilateral; CAMbi, continuous active motion bilateral; *d*, Cohen’s *d* effect size; *p*, probability value; iMVT, isometric maximal voluntary torque; EDC, epidural catheter; NFB, nerve femoral block; CRP, C-reactive protein.

^++^ Denotes a medium effect (Cohen’s *d* 0.50 to 0.79).

^+++^ Denotes a large effect (Cohen’s *d* ≥ 0.80).

* Denotes a significant difference between groups (*p* ≤ 0.025).

^†^ Denotes a statistical tendency towards a significant difference between groups (*p* ≤ 0.050).
